# The changing immune landscape of innate‐like T cells and other innate cells throughout life

**DOI:** 10.1111/imcb.70070

**Published:** 2025-12-10

**Authors:** Marziyeh Taheri, Christopher Menne, Jeremy Anderson, Louis Perriman, Shuo Li, Stuart P Berzins, Paul V Licciardi, Thomas M Ashhurst, Sedigheh Jalali, Daniel G Pellicci

**Affiliations:** ^1^ Murdoch Children's Research Institute Melbourne VIC Australia; ^2^ Department of Microbiology and Immunology, Peter Doherty Institute for Infection and Immunity University of Melbourne Melbourne VIC Australia; ^3^ Fiona Elsey Cancer Research Institute Ballarat VIC Australia; ^4^ Federation University Ballarat VIC Australia; ^5^ Department of Paediatrics University of Melbourne Melbourne VIC Australia; ^6^ Sydney Cytometry Core Research Facility The University of Sydney and Centenary Institute Sydney NSW Australia; ^7^ Faculty of Medicine and Health, School of Medical Sciences The University of Sydney Sydney NSW Australia; ^8^ Melbourne Centre for Cardiovascular Genomics and Regenerative Medicine Melbourne VIC Australia

**Keywords:** aging, CD markers, immunophenotyping, innate cells, innate‐like T cells, spectral flow cytometry

## Abstract

Spectral flow cytometry is an advanced immunological tool that can enable comprehensive analysis of the immune system by simultaneously comparing innate and adaptive immune cells. Here, using a 40‐color antibody panel, we advance our knowledge of innate‐like cells by investigating chemokine receptors and maturation markers not usually assessed on these populations, examining age‐related effects on these immune cell subsets. We characterize phenotypic changes of peripheral blood mononuclear cells (PBMCs) in three age groups: newborns (cord blood), adults aged 20–30 years, and adults aged > 70 years. We compare the age‐related changes of innate cells, including ILCs, NK cells, monocytes, dendritic cells, and innate‐like T cells, comparing them with memory T cells. We also examine subsets of CD4^−^CD8^−^ double‐negative (DN) T cells and CD3^+^CD161^+^ T cells, revealing they are phenotypically similar to known subsets of innate‐like T cells, and they also increase in frequency in an age‐related manner. The frequencies of ILC1 increased with age, ILC2 remained stable, whereas ILC3 peaked in young adults, and were higher than cord and older adults. Notably, we identify the NK cell maturation marker, CD57, as a universal marker that defines aging populations of both innate and adaptive immune cells. This study enhances our understanding of the ontogeny of human immune cells, highlighting significant age‐related changes in the frequency and phenotype of innate‐like populations of immune cells.

## Graphical abstract

The phenotype of innate‐like T cells changes with age. Here we describe these changes in relation to other subsets of innate cells.

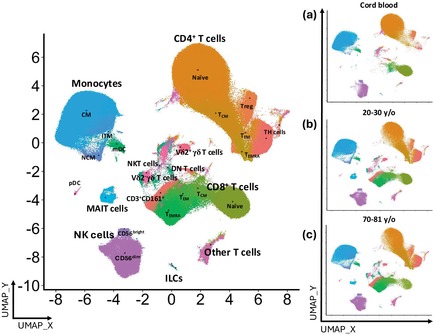

## INTRODUCTION

The immune system is made up of a network of cells that have substantial diversity in their frequency, phenotype and function. The immune system can be influenced by various factors such as age, sex, and disease.^1^, ^2^, ^3^, ^4^ Therefore, having a detailed understanding of the healthy immune system throughout life may help identify changes caused by human disease. Broadly, the immune system is subdivided into two arms, innate and adaptive, although innate‐like T cells exhibit features of both arms and play important roles in human immunity.^5^, ^6^, ^7^ Research on age‐related alterations of the immune system has been ongoing for many decades with a focus on T cell and B cell subsets and their responses,^8^, ^9^, ^10^, ^11^ although less is known about how subsets of innate cells change throughout life. This includes detailed analysis of NK cells, that is, CD56^dim^ NK cells, CD56^bright^ NK cells, in addition to dendritic cells (DCs), monocytes and innate lymphoid cells (ILCs). Furthermore, innate‐like T cells, including natural killer T (NKT) cells, mucosal‐associated invariant T (MAIT) cells, gamma delta (γδ) T cells, and subsets of double‐negative (DN) T cells and CD3^+^CD161^int^ T cells.

Recent advancements in multi‐omics methodologies and spectral flow cytometry have facilitated the simultaneous analysis of a large number of immune cell populations within a given biological sample.^4^, ^12^, ^13^, ^14^, ^15^ The inclusion of many markers in one antibody cocktail permits deeper analysis of human immune cells and provides the opportunity to potentially discover new subsets of immune cells. These studies offer an unprecedented depth to the exploration of novel combinations of markers and cell subsets, for example, the analysis of innate markers (CD57, CD16, NKG2A) on T cells, and the analysis of T cell markers (CD27, CD38, chemokine receptors) on innate cell subsets.

To shed further light on the intricacies of age‐related changes to innate‐like populations of immune cells, we employed spectral flow cytometry to phenotypically profile blood immune cells in three healthy age groups: (i) newborns (cord blood), (ii) young adults (20–30 years old), and (iii) old adults (70–81 years old). We previously demonstrated profound changes to the composition of peripheral blood cells from infancy to adulthood.^4^ While our focus was on the developmental trajectory beyond infancy, particularly childhood and schooling age, the current study includes immune profiling of cord blood that is less likely to have been exposed to environmental influences like microbial pathogens and childhood vaccines. The immune cells from cord blood exhibit an immature phenotype and have a reduced capacity to proliferate and secrete fewer cytokines.^16^ Whereas with age, the immune system undergoes immunosenescence, rendering older individuals more susceptible to various diseases, particularly cancer and infections.^17^, ^18^ This study utilizes a 40‐color antibody panel to dive deeper into the complexities of innate and adaptive cells, with a focus on innate‐like T cells that bridge the gap between these two arms of the immune system. Further, we examine ILC subsets (ILC1, 2, 3) which lack both T‐cell and B‐cell receptors (TCR and BCR) to recognize specific antigens but mirror helper T (Th) cells in their functional roles and profiles. For example, ILC1s are similar to Th1 cells in their ability to produce key pro‐inflammatory cytokines, ILC2s reflect the functions of Th2 cells through their responses associated with allergy and inflammation, and ILC3s resemble Th17 cells in their role in mediating responses related to mucosal immunity and inflammation.^19^, ^20^, ^21^ ILCs have recently been described in cord blood, peripheral blood,^20^, ^22^, ^23^, ^24^ as well as mucosal and barrier tissues^20^, ^21^, ^22^, ^25^; however, how ILC phenotypes change throughout life is less well understood. Our work explores how age‐related changes to innate‐like T cells (NKT cells, MAIT cells, and γδ T cells) compare to changes in innate and adaptive cells, including a detailed analysis of DN T cells and CD3^+^CD161^int^ T cells, which undergo changes in frequency and phenotype throughout life.

## RESULTS

**Figure 1:**
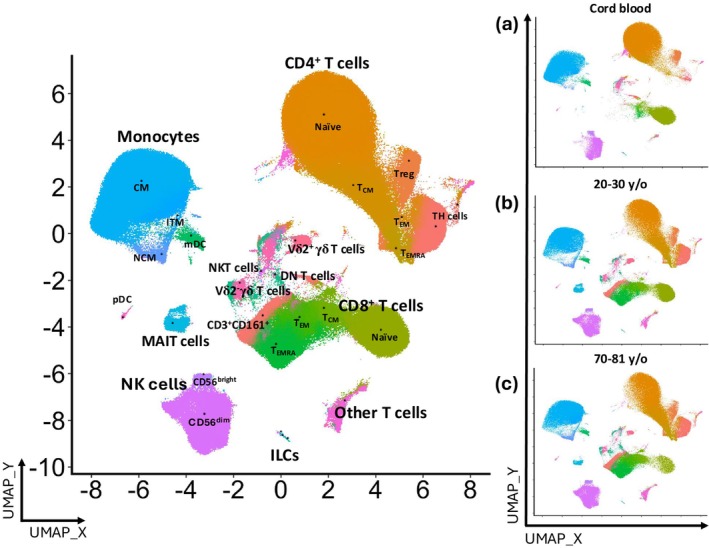
UMAP visualization of immune cell changes from cord blood and adult peripheral blood. (Left) A UMAP plot shows 25 manually annotated cell subsets from 24 blood donors identified by FLOWSOM clustering. Manual annotations were based on canonical markers shown in Supplementary figure 1. (Right) UMAP plots highlighting the changes in the immune cell populations of **(a)** cord blood, **(b)** young adults (20–30 years old), and **(c)** old adults (70–81 years old).

To investigate age‐related changes to innate cells and innate‐like T cells, we analyzed the immune system of healthy donor cord blood mononuclear cells (CBMCs) and healthy adult peripheral blood mononuclear cells (PBMCs) that were clustered into two age groups: younger adults aged 20–30 years and older adults aged 70–81 years (Supplementary table 1). We employed a 40‐color flow cytometry panel modified from our recent studies^4^, ^26^ that facilitates the simultaneous identification of several immune cell lineages. In addition to lineage markers to decipher the majority of T cell, B cell, NK cell, ILC, innate‐like T cell, monocyte, and dendritic cell subsets, we included several chemokine receptors and cell lineage and differentiation markers (Supplementary tables 2 and 3). To accommodate for the high complexity of the data generated and the plethora of distinct immune cell subsets, we performed an unsupervised high‐dimensional integration and analysis workflow using the Spectre toolkit in R, presenting our data using Uniform Manifold Approximation and Projection (UMAP) (Figure 1 and Supplementary figure 1).^27^ There were differences in the immune cell composition between CBMCs and PBMCs. The most striking differences were the near absence of CD4 and CD8 memory T cells from cord blood as well as the decrease of naïve T cells throughout life (Figure 1). Moreover, innate‐like T cells and NK cells were drastically increased in the two adult age groups, compared to cord blood (Figure 1 and Supplementary figure 1). Overall, this defines the immune status of the immune system in newborns, as well as in healthy adults and allows for the identification of age‐related changes in immune cell composition. We then further characterized innate cells and innate‐like T‐cell subsets using chemokine and differentiation markers expressed by innate and adaptive immune cells.

### Identification of innate lymphoid cells (ILCs) from cord and adult blood

**Figure 2:**
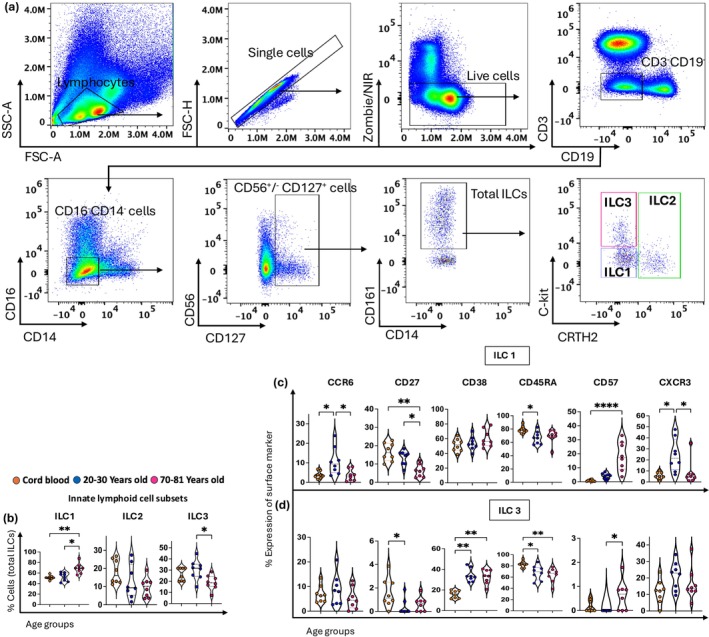
The frequency and phenotype of different subsets of innate lymphoid cells (ILCs) throughout life. **(a)** Flow cytometry plots from one cord blood mononuclear cell (CBMC) sample show a gating strategy for defining ILC1, ILC2 and ILC3: ILCs are gated from viable, single CD3^−^CD19^−^ T cells and subsequently gated for CD16^−^CD14^−^CD56^+/−^CD127^+^CD161^+^ cells. Then, ILC1, ILC2, and ILC3 subsets are defined using c‐Kit and CRTH2. **(b)** The violin plots show the age‐related distribution of ILC1, ILC2, and ILC3 from total ILC cells within three age groups: cord blood (*n* = 8, orange circles), young adults aged 20–30 years old (*n* = 8, blue circles), and old adults aged 70–81 years old (*n* = 8, pink circles). **(c, d)** The violin plots represent the percentage of CCR6^+^, CD27^+^, CD38^+^, CD45RA^+^, CD57^+^, and CXCR3^+^ cells within the ILC1 and ILC3 subsets. Data are shown with the median. Each dot represents data from one donor, and each color represents one age group. A nonparametric Kruskal–Wallis test with Dunn's multiple comparisons test was used to compare all three groups. *P*‐values are *P* > 0.05 (ns), *P ≤ 0.05; **P ≤ 0.01; ****P ≤ 0.0001.

First, we determined the frequency of ILC1, ILC2, and ILC3 subsets from total ILC cells, gated as CD3^−^CD19^−^CD14^−^CD16^−^CD127^+^CD56^−/+^CD161^+^ cells (Figure 2a). ILC subsets were defined by their differential expression of CD117 (c‐Kit) and CD294 (CRTH2) from cord blood, young adult blood, and older adult blood (Figure 2a, b and Supplementary figure 2). ILC1 was the dominant ILC subset, comprising approximately 50–60% of total ILCs, while ILC2 was the rarest (~10–15%). All ILC subsets displayed distinct patterns, with ILC1 frequencies increasing with age, ILC2 remaining relatively stable with age, while ILC3 peaked in young adults and were higher than cord and older adult blood (Figure 2b). Given the frequency of ILC2 was low across all age groups, it precluded further downstream analysis of these cells. Phenotypic analysis of ILC1 indicated that CCR6 expression was highest (~10%) in young adults and low in cord and older adult blood (~4%) (Figure 2c and Supplementary figure 2). CD27 was expressed by ILC1 from cord blood (~16%) and decreased with age (~6%). An age‐related decrease was observed for the expression of CD45RA by both ILC1 and ILC3 (from ~80% to ~60%) (Figure 2c, d and Supplementary figure 2). The proportion of CD38^+^ ILC1 and ILC3 increased with age, particularly for ILC3, and CD57 was highest on ILC1 cells from older adults (Figure 2c, d and Supplementary figure 2). CXCR3 expression on ILC3 was stable across the three age groups (10–20%) but was significantly higher on ILC1 from young adults compared to cord and older adult blood (Figure 2c, d and Supplementary figure 2).

### Characterization of innate‐like T cells

**Figure 3:**
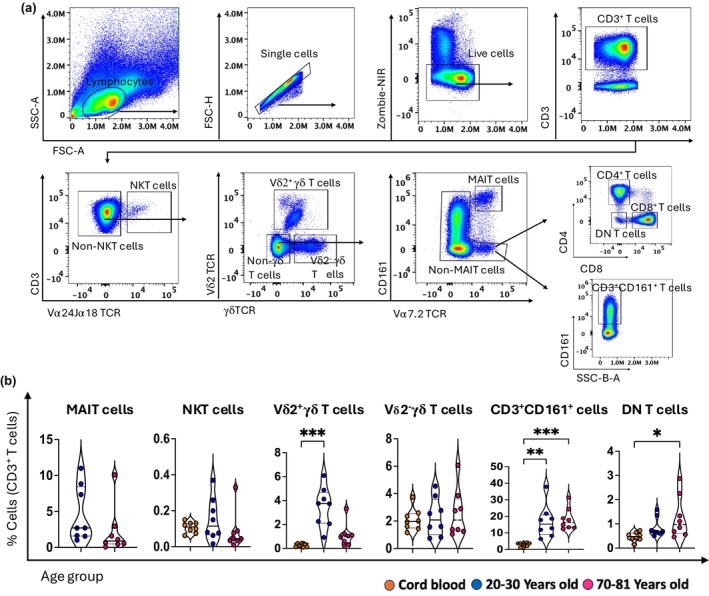
The frequency of innate‐like T cells changes with age. **(a)** Flow cytometry plots of one young adult PBMC sample showing a gating strategy for defining different subsets of unconventional T cells, including mucosal‐associated invariant T (MAIT) cells, Vδ2^+^ and Vδ2^−^ gamma delta (γδ) T cells, natural killer T (NKT) cells, CD3^+^CD161^int^ T cells and double‐negative (DN) T cells. **(b)** The violin plots show the age‐related distribution of MAIT, NKT, Vδ2^+^ and Vδ2^−^ γδ T, CD3^+^CD161^int^ T, and DN T cells within three age groups: cord blood (*n* = 8, orange circles), young adults aged 20–30 years (*n* = 8, blue circles), and old adults aged 70–81 years (*n* = 8, pink circles). Data are shown with the median. Each dot represents data from one donor, and each color represents one age group. A nonparametric Kruskal–Wallis test with Dunn's multiple comparisons test was used to compare all three groups. The Mann–Whitney *U*‐test (nonparametric) was used for pairwise comparison between young and older adults in MAIT cell analysis. *P*‐values are *P* > 0.05 (ns), *P ≤ 0.05; **P ≤ 0.01; ***P ≤ 0.001.

MAIT cells could not be reliably detected in cord blood using antibodies towards TCR Vα7.2 and CD161, and thus, MAIT cells from cord blood were not included in our analysis.^28^ MAIT cells and Vδ2^+^ γδ T‐cell frequency were highest in young adult blood, with Vδ2^+^ γδ T cells being barely detectable in cord blood (Figure 3a, b). As previously reported, both MAIT cells and Vδ2^+^ γδ T cells declined with age,^4^, ^28^, ^29^ although the presence of an outlier for MAIT cells in older adult blood prevented significance (Figure 3a, b). Interestingly, the frequency of NKT cells and Vδ2^−^ γδ T cells remained unchanged across all age groups (Figure 3a, b). After exclusion of these known subsets of innate‐like T cells (NKT, MAIT, and γδ T cells), we observed a population of CD3^+^ T cells that expressed CD161 (Figure 3a, b), and we tentatively suggest these cells represent another population of innate‐like T cells. These CD3^+^CD161^+^ T cells expressed intermediate (int) levels of CD161 compared to MAIT cells (Figure 3a).^30^ The frequency of CD3^+^CD161^int^ T cells in cord blood was ~3% but made up ~16% of CD3^+^ T cells in adult blood (Figure 3b). Similarly, after the exclusion of NKT cells, MAIT cells and γδ T cells, we also examined CD4^−^CD8^−^ T cells, herein defined as DN T cells (Figure 3a) and revealed an age‐related increase in their frequency between cord blood (~0.3%) and adults (~1%) (Figure 3b).

**Figure 4:**
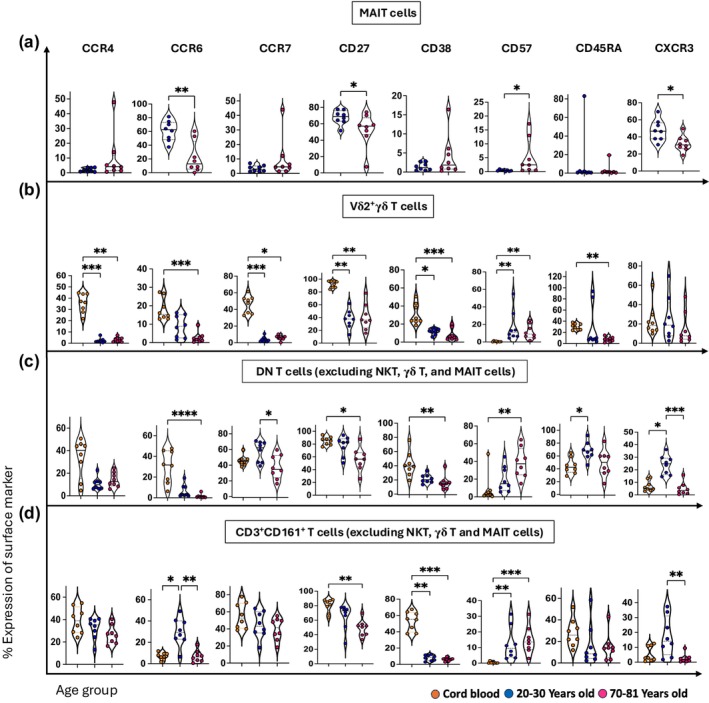
Comparison of cell surface marker expression indicates similarities between MAIT, Vδ2^+^ γδ T, DN T and CD3^+^CD161^int^ T cells. **(a–d)** Violin plots represent the age‐related distribution of CCR4^+^, CCR6^+^, CCR7^+^, CD27^+^, CD38^+^, CD57^+^, CD45RA^+^ and CXCR3^+^ cells on **(a)** MAIT cells, **(b)** Vδ2^+^ γδ T, **(c)** DN T cells, and **(d)** CD3^+^CD161^int^ T cells. High‐dimensional flow cytometry was carried out on blood samples from three age groups: cord blood (*n* = 8, orange circles), young adults aged 20–30 years old (*n* = 8, blue circles), and older adults aged 70–81 years old (*n* = 8, pink circles). Each dot represents data from one donor, and each color represents one age group. A nonparametric Kruskal–Wallis test with Dunn's multiple comparisons test was used to compare all three groups. The Mann–Whitney *U*‐test (nonparametric) was used for pairwise comparison between young and older adults in MAIT cell analysis. *P*‐values are *P* > 0.05 (ns), *P ≤ 0.05; **P ≤ 0.01; ***P ≤ 0.001; ****P ≤ 0.0001.

To further understand the complexities of human innate‐like T‐cell subsets, we compared the phenotype of MAIT cells and Vδ2^+^ γδ T cells with CD3^+^CD161^int^ T cells and DN T cells using a range of cell surface markers (Figure 4a–d and Supplementary figures 3–6). Interestingly, CCR4, CCR7, and CD38 were highly expressed by Vδ2^+^ γδ T cells, DN T cells, and CD3^+^CD161^int^ T cells from cord blood (Figure 4b–d and Supplementary figures 4–6). CCR4 and CD38 were decreased in an age‐related manner by Vδ2^+^ γδ T cells, CD3^+^CD161^int^ T cells, and DN T cells (Figure 4b–d and Supplementary figures 4–6). The expression of CCR7 and CD45RA showed an age‐related decrease in Vδ2^+^ γδ T cells and CD3^+^CD161^int^ T cells, whereas DN T cells exhibited the highest expression levels of both molecules in young adults relative to other age groups (Figure 4b–d and Supplementary figures 4–6). Vδ2^+^ γδ T cells, DN T cells and CD3^+^CD161^int^ T cells expressed high levels of CD27 in cord blood (~90%) and showed an age‐related decrease which dropped to ~40–50% in older adults (Figure 4b–d and Supplementary figures 4–6). A similar age‐related reduction in CD27 expression was also observed for MAIT cells between the two adult groups (Figure 4a and Supplementary figure 3). Notably, CD57 expression typically increased on innate‐like T cells from cord blood to older adults, and it was the highest on DN T cells from the older adult group (~40%) (Figure 4a–d and Supplementary figures 3–6). CCR6 expression, which is often used to define type III effector populations of Vδ2^+^ γδ T cells,^31^ was similar between Vδ2^+^ γδ T cells and DN T cells, and showed an age‐related decrease from cord to adult blood (from ~20% to ~2%). CCR6 on MAIT cells and CD3^+^CD161^int^ T cells was similar in adult blood, representing ~30–60% of cells in young adults, before decreasing in older adults (~10%) (Figure 4a–d and Supplementary figures 3–6). CXCR3 expression was highest in young adults in MAIT cells, Vδ2^+^ γδ T cells, CD3^+^CD161^int^ T cells, DN T cells (Figure 4a–d and Supplementary figures 3–6), as well as for Vδ2^−^ γδ T cells (Supplementary figure 7). We also observed an age‐related decrease in the expression of CD27, CD38, CCR7 as well as an age‐related increase in the expression of CCR4 and CD57 on Vδ2^−^ γδ T cells (Supplementary figure 7).

### Innate‐like T‐cell subsets share phenotypic properties with memory subsets of conventional T cells

**Figure 5:**
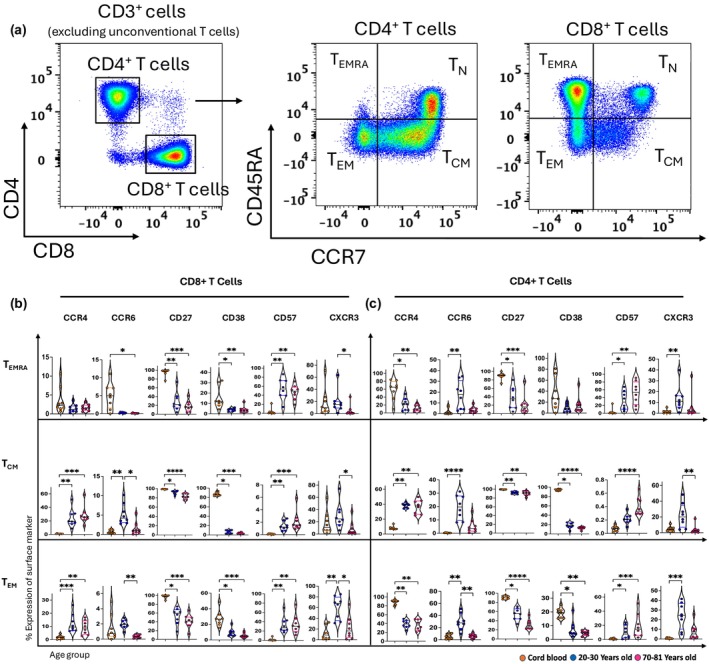
Memory subsets of conventional CD4^+^ T cells and CD8^+^ T cells exhibit some similar characteristics to innate‐like T cells. **(a)** Flow cytometry plots of one young adult PBMC sample showing the gating strategy for defining different memory subsets of CD4^+^ T cells and CD8^+^ T cells after excluding NKT cells, γδ T cells, and MAIT cells. **(b, c)** Violin plots illustrate the age‐related distribution of CCR4^+^, CCR6^+^, CD27^+^, CD38^+^, CD57^+^, and CXCR3^+^ cells in T central memory (T_CM_), T effector memory (T_EM_), and T effector memory CD45RA^+^ (T_EMRA_) CD8^+^ T cells and CD4^+^ T cells within three age groups: cord blood (*n* = 8, orange circles), young adult (*n* = 8, 20–30 years old) (blue circles), and old adults (*n* = 8, 70–81 years old) (pink circles). The data are presented with the median. Each dot represents data from one donor, and each color represents one age group. A nonparametric Kruskal–Wallis test with Dunn's multiple comparisons test was used to compare all three groups. *P*‐values are as follows: *P* > 0.05 (ns), *P ≤ 0.05; **P ≤ 0.01; ***P ≤ 0.001; ****P ≤ 0.0001.

A defining feature of human innate‐like T cells is that they express maturation/memory markers that are typically acquired during development in the thymus.^32^, ^33^, ^34^ To determine if there were any similarities between the phenotype of innate‐like T cells and CD4^+^ and CD8^+^ memory T‐cell subsets, we compared the expression of surface markers of innate‐like T cells to effector memory T cells (T_EM_), central memory T cells (T_CM_) and effector memory T cells expressing CD45RA (T_EMRA_) (Figure 5a–c). Consistent with our findings of innate‐like T cells (Figure 4), we detected an age‐related decrease of CD27 and CD38 expression and an increase in CD57 expression across all memory subsets of CD4^+^ and CD8^+^ T cells (Figure 5b, c). Moreover, CD38 was highly expressed by CD4^+^ and CD8^+^ T_CM_ cells from cord blood (~90%), while other T‐cell subsets (T_EM_, T_EMRA_, Vδ2^+^ γδ T cells and DN T cells) from cord blood had moderate expression of CD38 (~10–20%) (Figures 4b, c and 5b, c and Supplementary figures 8 and 9). CXCR3 expression was typically highest among innate‐like T cells and memory subsets of CD4^+^ and CD8^+^ T cells from young adults, compared to cord and older adult blood (Figures 4 and 5b, c and Supplementary figures 3–9). Vδ2^+^ γδ T cells from cord blood had high frequencies of CCR4^+^ cells (~40%) compared to adults (~5%) (Figure 4), and a similar trend was seen for CCR4 expressed by CD4^+^ T_EMRA_ (from 60% to 10%) and CD4^+^T_EM_ cells (from 90% to 40%) (Figure 5c). In contrast, CCR4 increased with age on CD4^+^ T_CM_, CD8^+^ T_CM_ and CD8^+^ T_EM_ cells (Figure 5b, c). Notably, the expression of CCR6 showed an age‐related decrease by CD8^+^ T_EMRA_ cells, similar to Vδ2^+^ γδ T cells and DN T cells (Figures 4a–c and 5b and Supplementary figure 9). Other subsets of memory T cells trended similarly to MAIT cells and CD3^+^CD161^int^ T cells, where the highest expression of CCR6 was observed in young adult blood (Figures 4a, d and 5b, c and Supplementary figures 8 and 9).

### Vδ2^+^ γδ T cells and NK cells undergo similar age‐related changes in their phenotype

**Figure 6:**
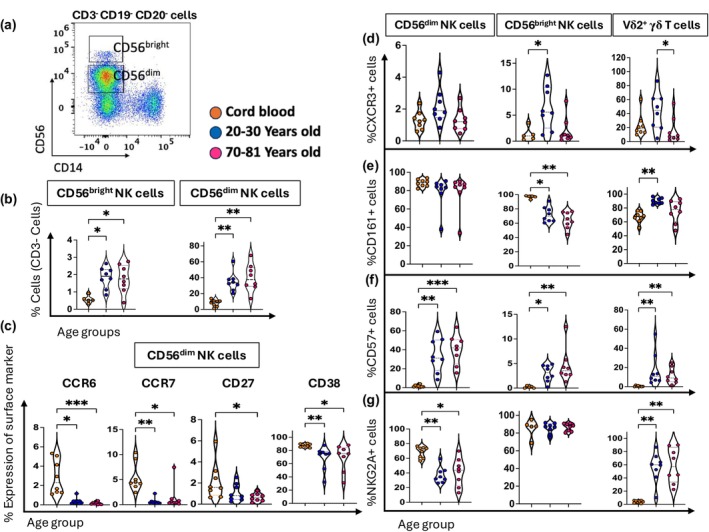
The phenotypic changes of Vδ2^+^ γδ T cells exhibit similar characteristics to CD56^dim^ and CD56^bright^ NK cells. **(a)** Flow cytometry plots of one young adult PBMC sample showing the gating strategy for defining CD56^dim^ and CD56^bright^ NK cells. **(b)** Violin plots illustrate the age‐related distribution of CD56^dim^ NK and CD56^bright^ NK cells within three age groups: cord blood (*n* = 8, orange circles), young adult (*n* = 8, 20–30 years old) (blue circles), and old adults (*n* = 8, 70–81 years old) (pink circles). **(c)** Violin plots depict the proportion of CCR6^+^, CCR7^+^, CD27^+^, CD38^+^ CD56^dim^ NK cells. **(d–g)** Violin plots show the proportion of **(d)** CXCR3^+^ cells, **(e)** CD161^+^ cells, **(f)** CD57^+^ cells, **(g)** NKG2A^+^ cells comparing CD56^dim^ NK cells, CD56^bright^ NK cells and Vδ2^+^ γδ T cells in the three age groups. Data are shown with the median. Each dot represents data from one donor, and each color represents one age group. A nonparametric Kruskal–Wallis test with Dunn's multiple comparisons test was used to compare all three groups. *P*‐values are *P* > 0.05 (ns), *P ≤ 0.05; **P ≤ 0.01; ***P ≤ 0.001.

Vδ2^+^ γδ T cells and NK cells share similar phenotypes and functions including the secretion of pro‐inflammatory cytokines and cytotoxic killing granules.^32^, ^35^, ^36^, ^37^, ^38^ Therefore, we closely examined the frequencies and phenotypes of two subsets of NK cells: CD56^dim^ and CD56^bright^ (Figure 6a) and compared them to Vδ2^+^ γδ T cells (Figures 4b and 6 and Supplementary figure 10). The frequency of CD56^bright^ and CD56^dim^ NK cells increased in adults (~2% and ~35%, respectively) compared to their frequencies in cord blood (~0.5% and ~10%, respectively) (Figure 6b). Analysis of the expression of chemokine receptors and classic T cell surface markers on CD56^dim^ and CD56^bright^ NK cells revealed that CCR6, CCR7, CD27, and CD38 expression decreased with age on CD56^dim^ NK cells (Figure 6c–g), akin to that observed for Vδ2^+^ γδ T cells (Figure 4b). Moreover, among Vδ2^+^ γδ T cells, CD56^dim^ and CD56^bright^ NK cells, the frequency of CXCR3^+^ cells appeared highest in young adults (Figure 6d).

Although the expression of CD161 on both subsets of NK cells was high from cord blood (~90%), an age‐related decrease was only observed for CD56^bright^ NK cells (~60%), and it was highest on Vδ2^+^ γδ T cells in young adults (Figure 6e). While CD161 decreased on CD56^bright^ NK cells, the NK cell maturation marker, CD57, increased on NK subsets and Vδ2^+^ γδ T cells (Figure 6f). Specifically, CD57 was higher on CD56^dim^ NK cells (~30%) compared to CD56^bright^ NK cells (~3%) and Vδ2^+^ γδ T cells (~10%) in adult blood samples (Figure 6f). NK cells express high levels of the inhibitory receptor, NKG2A.^39^, ^40^, ^41^ We reveal this marker decreases on CD56^dim^ NK cells over time (Figure 6g) compared to CD56^bright^ NK cells, while, notably, an age‐related increase was observed for Vδ2^+^ γδ T cells (Figure 6g and Supplementary figure 10).

### Age‐related changes to the frequency and phenotype of the innate myeloid cell compartment

**Figure 7:**
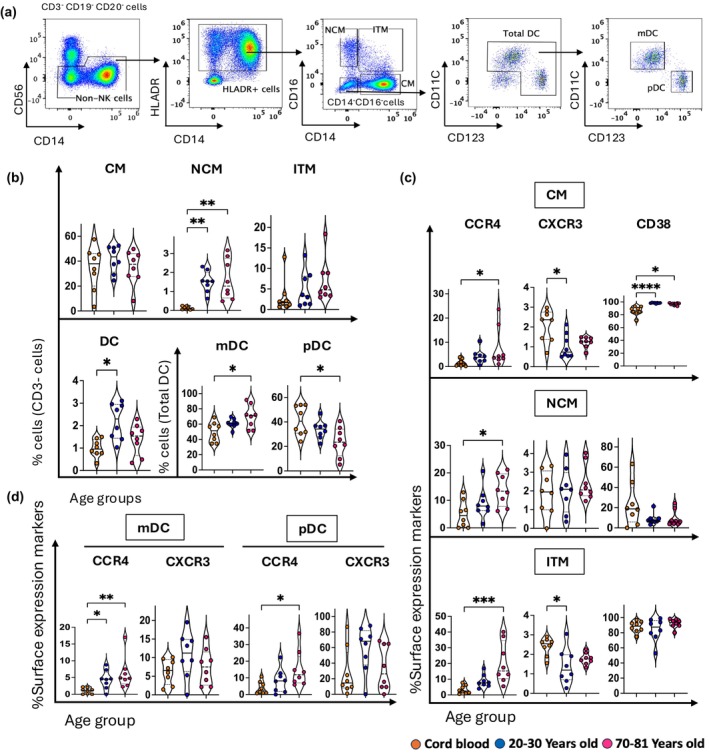
Analysis of monocytes and dendritic cell subsets from cord blood and adult blood illustrated varied age‐related alterations. **(a)** Flow cytometry plots of one young adult PBMC sample showing the gating strategy for defining classical (CM), nonclassical (NCM), intermediate (ITM) monocytes, total dendritic cells (DCs), myeloid DCs (mDC), and plasmacytoid DCs (pDC). **(b)** Violin plots illustrate the age‐related distribution of CM, NCM, ITM, DCs, mDC, and pDC within three age groups: cord blood (*n* = 8, orange circles), young adult (*n* = 8, 20–30 years old) (blue circles), and old adults (*n* = 8, 70–81 years old) (pink circles). **(c)** Violin plots represent the age‐related distribution of CCR4^+^, CXCR3^+^, and CD38^+^ cells on CM, NCM, and ITM in the three age groups. **(d)** Violin plots represent the age‐related distribution of CCR4 and CXCR3 on mDC and pDC cells in the three age groups. Data are shown with the median. Each dot represents data from one donor, and each color represents one age group. A nonparametric Kruskal–Wallis test with Dunn's multiple comparisons test was used to compare all three groups. *P*‐values are *P* > 0.05 (ns), *P ≤ 0.05; **P ≤ 0.01; ***P ≤ 0.001; ****P ≤ 0.0001.

To examine how the innate myeloid compartment comprising of monocytes and dendritic cells alters throughout life, we examined the frequencies and phenotypes of classical monocytes (CM), intermediate monocytes (ITM), and nonclassical monocytes (NCM), in addition to total dendritic cells (DCs), myeloid DCs (mDCs), and plasmacytoid DCs (pDCs) (Figure 7a, b). The frequency of CM and ITM remained relatively unchanged across the different age groups, whereas NCM were barely detectable in cord blood and increased with age (Figure 7b). The frequency of total dendritic cells (DCs) was highest in young adults (~2%) (Figure 7b). Separation of DCs into mDCs and pDCs subsets reveals that mDCs were highest in older adult blood, and conversely, pDCs were highest in cord blood (Figure 7b).

Phenotypic analysis of different subsets of monocytes (Figure 7c and Supplementary figure 11) and dendritic cells (Figure 7d and Supplementary figure 12) shows an age‐related increase in CCR4 on all of these cell types. This was consistent with the results for some T‐cell memory subsets (CD4^+^ T_CM_, CD8^+^ T_CM_, CD8^+^ T_EM_) (Figure 5); however, other T‐cell memory subsets and all innate‐like T cells show a decrease in CCR4 expression with age (Figures 4 and 5). While our work reveals that CD38 is downregulated on NK cells, innate‐like T cells and conventional memory T cells in an age‐dependent manner (Figures 4b–d, 5b, c and 6c), CD38 expression increased on CM throughout life (Figure 7c). The expression of CXCR3 appeared higher on mDC and pDC from young adults, although this was not significant (Figure 7d and Supplementary figure 12). Similar trends were seen for CD56^dim^ and CD56^bright^ NK cells and Vδ2^+^ γδ T cells (Figure 6d). Unlike other cell types, monocytes did not display a major population of CXCR3^+^ cells (Figure 7c and Supplementary figure 11).

### Sex‐dependent variation in the frequency and phenotype of innate‐like cells across age groups

To address possible gender‐related variability in immune cell subsets above, we analyzed male and female participants separately within the two adult age groups (no gender was recorded for cord blood donors). While no major differences were observed, some minor differences were identified. Specifically, men had a higher proportion of CD56^dim^ NK cells from young adults, and older men also had a higher frequency of Vδ2^+^ γδ T cells, compared to age‐matched women (Supplementary figure 13a). In addition, young adult men had higher proportions of CCR6^+^ and CD57^+^ ILC1, CCR6^+^ ILC3, and CCR4^+^ CD4^+^ T_EMRA_ cells but lower frequencies of CD38^+^ Vδ2^−^ γδ T cells and CCR7^+^ DN T cells, compared to age‐matched women (Supplementary figure 13b).

## DISCUSSION

Spectral flow cytometry is continuing to advance at a rapid rate, allowing for superior analysis of the immune system. This technology could allow for the identification of subsets of cells that have not yet been defined and that may have important roles in human immunity. Here, we used a 40‐color flow cytometry panel that includes a broad range of cell surface markers to profile immune cell subsets in human blood. This approach gave us the opportunity to identify and characterize immune cell populations, including rare subsets, for more comprehensive analysis. For example, in this study, after excluding known subsets of innate‐like T cells (NKT, γδ T, and MAIT cells), we focused on the remaining CD3^+^ T cells to identify DN T cells as well as a large population of cells that express intermediate levels of CD161 (CD161^int^). Many studies have analyzed DN T cells from healthy human blood; however, they did not exclude known subsets of innate‐like T cells.^42^, ^43^, ^44^ Traditionally, it has been reported that DN T cells could be either αβTCR^+^ cells or γδTCR^+^ cells,^45^ while our main focus was on DN T cells that were not γδT cells, enabling a more accurate comparison of the similarities and differences between these subsets of cells. For example, a previous study reported a significant decrease in the frequency of blood DN T cells in older adults aged between 51 and 80 years,^43^ which may be attributable to a decrease in innate‐like T cells, particularly Vδ2^+^ γδ T cells that are predominantly DN.^32^, ^45^ By excluding innate‐like T cells, we observed the opposite trend, whereby the frequency of DN T cells increases with age. Notably, we observed the same increase in CD3^+^CD161^int^ T cells.

Using a comprehensive immune phenotyping panel, we have thoroughly investigated the effects of age on the immune system in blood from healthy donors by comparing newborns' immune system (using cord blood) with young adults and elderly people, including the analysis of chemokine receptors, naive and memory markers. The phenotypic analysis of DN T cells revealed that the expression of CCR4, CCR6, CD27, CD38, CD57, and CXCR3 on these cells is strikingly similar to MAIT and Vδ2^+^ γδ T cells within each age group, although CD45RA remained relatively high on DN T cells from adult blood, while it was low on MAIT and Vδ2^+^ γδ T cells. These findings suggest that DN T cells (or subsets thereof) may represent a currently undefined subset of innate‐like T cells; therefore, further investigations are required to define their TCR repertoire, antigen specificity, transcriptional profile, function and ultimately their role in the human immune system. CD161^int^ T cells have been previously studied by the Klenerman group, which revealed that distinct subsets of CD161^int^ T cells share a conserved transcriptional signature with other innate‐like T‐cell subsets and can be activated by IL‐12 and IL‐18.^46^, ^47^ Furthermore, the same group revealed that CD8^+^CD161^int^ T cells shared phenotypic and functional characteristics typical of innate‐like T cells, including the expression of the transcription factors PLZF, Tbet, and Eomes; cytotoxic killing granules perforin and granzyme B; and cytokines IFN‐γ and IL‐2.^48^ Interestingly, CD8^+^CD161^int^ T cells included cells specific to viral peptides that are not usually recognized by innate‐like T cells.^48^ Although CD3^+^CD161^int^ T cells share an overlapping phenotype with Vδ2^+^ γδ T cells and DN T cells, their high CCR6 expression in young adults more closely resembles that of MAIT cells as well as memory CD4^+^ and CD8^+^ T‐cell subsets.

Our study also found that innate‐like T cells share phenotypic similarities with ILC cells. For example, analysis of ILC1 and ILC3 showed that these cells had reduced CD27 and CD45RA in older adult blood compared to cord blood, and this was similar to our observations of MAIT cells and Vδ2^+^ γδ T cells. ILC1 and ILC3 subsets showed increased expression of CD57 with age, akin to other subsets of innate cells and innate‐like T cells in this study. These three markers are well‐established markers of T‐cell maturation and differentiation^49^, ^50^ but are less commonly described for ILC subsets. Interestingly, we observed the expression of CD38 increased on ILC3 in peripheral blood compared to cord blood, a trend that was also seen for classical monocytes. CD38 expression typically decreases on conventional memory T cells, innate‐like T cells and NK cells throughout life. Given the multitude of functions described for CD38 ranging from cell activation to migration and cytokine release,^51^, ^52^, ^53^ these findings suggest a unique role of CD38 expression in regulating ILCs and monocyte immune function. Collectively, our data reveal age‐related maturation and differentiation of ILCs, which may impact their ability to influence immune outcomes, although subsequent studies are needed to confirm this.

Previous studies have revealed similar phenotypic and functional characteristics between NK cells and γδ T cells, emphasizing how γδ T cells can bridge the innate and adaptive immune responses. For example, both cell types can produce cytokines following cell activation.^38^, ^54^, ^55^ They also exhibit dual effects in combating microbial and viral infections, cancerous cells and in graft‐versus‐host disease (GVHD).^38^, ^54^, ^55^, ^56^ We found that Vδ2^+^ γδ T cells shared higher phenotypic similarities with CD56^dim^ NK cells (CCR6, CCR7, CXCR3, CD27, CD38, and CD57) compared to CD56^bright^ NK cells. In this study, CD57, commonly considered a maturation marker for NK cells,^57^, ^58^, ^59^ was found to increase with age on Vδ2^+^ γδ T cells, reflecting a pattern similar to that previously reported in CD16^+^CD56^dim^ NK cells.^59^, ^60^, ^61^ High levels of CD57 on CD16^+^CD56^dim^ NK cells have been associated with increased cytotoxicity and responsiveness to CD16‐mediated stimulation, which is particularly interesting given human Vδ2^+^ γδ T cells can also express CD16. In contrast, CD57 expression on T cells is often viewed as a marker of immunosenescence, indicating a state of terminal differentiation with limited proliferative capacity.^62^, ^63^, ^64^, ^65^ This phenomenon is seen in healthy individuals but may also indicate virus‐specific T cells in viral infections, as well as CD57^+^ NK cells that expand in response to HCMV infection.^65^, ^66^, ^67^, ^68^ Further assessment is needed to understand how age‐related increases of CD57 expression might impact the function of Vδ2^+^ γδ T cells, other innate‐like T cells, and ILCs.

Interestingly, we observed that CD161 expression did not change on CD56^dim^ NK cells with age but decreased with age on CD56^bright^ NK cells, suggesting an age‐related decline in the pro‐inflammatory potential of CD56^bright^ NK cells.^69^ In contrast, CD161 expression on Vδ2^+^ γδ T cells was higher in young adults and lower in older adults, which might indicate an age‐related modulation in cytokine responsiveness. While CD57 and CD161 are both implicated in immune cell functionality, CD161 expression on NK cells was independent of CD57 expression, suggesting that CD161^+^ NK cells retain their pro‐inflammatory cytokine potential irrespective of CD57‐mediated maturation status. This independence between CD57 and CD161 highlights a functional distinction, with CD161 primarily marking a cytokine producing subset, whereas CD57 is linked with a more differentiated, “adaptive” NK cell phenotype. Together, these findings provide potential new insights into the age‐related and functional characteristics of NK and γδ T cells.

Altogether, our study underscores the dynamic nature of the immune system across different life stages, from the immature immune phenotype observed in cord blood to the aged, immunosenescent phenotype in older adults. By utilizing high‐dimensional spectral flow cytometry, we were able to identify and characterize age‐related phenotypic changes in innate‐like T cells, as well as innate cells. These findings not only deepen our understanding of immune development during life but also highlight the potential for identifying novel immune cell subsets important in human immunity. The role of CD161^int^ T cells and DN T cells represents two highly prevalent populations of immune cells that are poorly studied in the context of human disease that clearly warrant further exploration. Moreover, our research lays the foundation for further exploration into targeted therapies and personalized medicine, particularly in designing age‐specific vaccine strategies to enhance immune protection in the most vulnerable populations: newborns and the elderly.

## METHODS

### Ethics statement

Healthy adult blood was provided by the Australian Red Cross, Lifeblood Australia, agreement number 23‐06VIC‐01 and cord blood was provided by the Royal Children's Hospital, with ethics approval from the Royal Children's Hospital Melbourne Human Research Ethics Committee (HREC24131).

### Human blood samples

Peripheral blood mononuclear cells from healthy adult donors and cord blood mononuclear cells (CBMCs) from umbilical cord blood were isolated using Ficoll‐Paque (GE Healthcare, NSW, Australia) and preserved in freezing media consisting of 10% dimethyl sulfoxide (DMSO) (Sigma‐Aldrich, NSW, Australia) and 90% fetal bovine serum (FBS) (Sigma‐Aldrich, NSW, Australia). Cells were frozen at a rate of −1°C/min in a −80°C freezer using a Cool Cell (Corning, Arizona, USA) and then transferred to the vapor phase of liquid nitrogen (−196°C) for long‐term storage. A total of 24 samples were examined in this study: 8 CBMC samples and 16 adult PBMC samples (8 young adult and 8 older adult samples). For adult blood, each group contained an equal ratio of male and female individuals (Supplementary table 1).

### High‐dimensional flow cytometry analysis

A 40‐color antibody panel was used to assess the frequency of immune cell subsets (Supplementary table 2) in cord blood, young adult blood, and older adult blood, comparing their frequency and phenotypic properties. PBMC or CBMC samples were thawed and stained with an established antibody cocktail in three stages: (i) staining of chemokine receptors with antibodies in FACS buffer was performed at room temperature for 20 min followed by (ii) surface markers staining at 4°C for 20 min, and then (iii) Zombie NIR to identify any dead cells, with four additional surface markers at 4°C for another 20 min (Supplementary Table 3). Cells were washed between each step by removing the supernatant after 5 min of centrifugation at 400 g and resuspending the cell pellets with FACS buffer (PBS enriched with 2% FBS). Cells were analyzed using a 5‐laser Cytek Aurora (Cytek Biosciences, California, USA).

### Statistical analysis

FlowJo software (10.9.0 version; BD, USA) was used to apply a manual gating method, identifying all critical cell subsets of our experiment according to the gating strategy illustrated in Figures 2, 3, 4, 5, 6, 7 and Supplementary figures 2–12. Cell subsets with 50 events or fewer in flow cytometry were excluded from phenotypic analysis. To compare all three groups including cord blood, young and old blood from healthy adults, the nonparametric Kruskal–Wallis test with Dunn's multiple comparisons test was employed, and an adjusted *P*‐value < 0.05 was regarded as significant. The Mann–Whitney *U*‐test (nonparametric) was used for pairwise comparisons between young and older adults for MAIT cell analysis. A Mann–Whitney *U*‐test (nonparametric) was also used to compare the frequency of cells between female and male donors. Data were plotted using GraphPad Prism software version 10 (San Diego, CA, USA). In addition, we used the Spectre R toolkit to generate unsupervised t‐SNE plots for visualization of high‐parameter flow cytometry in Figure 1, as previously described.^4^, ^27^ Briefly, data were imported into R and merged into a single data table. Data were then transformed with per‐channel arcsinh values, using a co‐factor of 5000 for all channels. Data were then clustered with FlowSOM^70^ using a grid size of 14 × 14 and a set target of 40 metaclusters. Alternatively, phenotypic gates were applied based on prescribed levels of expression of multiple markers, derived from manual data exploration in FlowJo. Clustered/gated data were then randomly down‐sampled to 200 000 cells per group, and plotted using UMAP^71^ with default parameters. Lineages were then analyzed and manually annotated independently, before being re‐merged as a complete dataset. Residual granulocytes and cells with no expression of immune markers were filtered out for final analysis, which resulted in a dataset enriched for mononuclear immune cells.

## AUTHOR CONTRIBUTIONS

Marziyeh Taheri performed the research, carried out data analysis, figure preparation and drafted the initial version of the manuscript. Thomas M Ashhurst contributed to data preparation. Christopher Menne, Jeremy Anderson, Louis Perriman, Shuo Li, Stuart P Berzins, Paul V Licciardi and Thomas M Ashhurst contributed to project ideas and edited the manuscript. Sedigheh Jalali and Daniel G Pellicci concieved the study, revised the manuscript and supervised all aspects of the research.

## CONFLICT OF INTEREST

The authors declare no conflicts of interest.

## Supporting information


Supplementary table 1
Supplementary table 2
Supplementary table 3
Supplementary figure 1
Supplementary figure 2
Supplementary figure 3
Supplementary figure 4
Supplementary figure 5
Supplementary figure 6
Supplementary figure 7
Supplementary figure 8
Supplementary figure 9
Supplementary figure 10
Supplementary figure 11
Supplementary figure 12
Supplementary figure 13

## Data Availability

Most data in this study are present within the published article. Additional data and the R script are available by request to the corresponding authors.
