# Supplementary material for: The changing immune landscape of innate‐like T cells and other innate cells throughout life
Source: Immunol Cell Biol. 2025 Dec 10;104(1):74–88. doi: 10.1111/imcb.70070 (PMC12800730; doi:10.1111/imcb.70070)
Supplement: Supplementary file 1 — Supplementary table 1 Supplementary table 2 Supplementary table 3 Supplementary figure 1 Supplementary figure 2 Supplementary figure 3 Supplementary figure 4 Supplementary figure 5 Supplementary figure 6 Supplementary figure 7 Supplementary figure 8 Supplementary figure 9 Supplementary figure 10 Supplementary figure 11 Supplementary figure 12 Supplementary figure 13 [file IMCB-104-74-s001.docx]

**Supplementary table 1.** Demographic data of the cord blood and peripheral blood samples.

| Groups | Age range | No. of samples/Gender |
| --- | --- | --- |
| Group-1 | Cord blood (newborns) | n: 8 (gender not specified) |
| Group-2 | 20-30 years old (young adults) | n: 8 (Female: 4/ Male: 4) |
| Group-3 | 70-81 years old (old adults) | n: 8 (Female: 4/ Male: 4) |

**Supplementary table 2.** Immune cell markers for cord blood mononuclear cells (CBMCs) and peripheral blood mononuclear cells (PBMCs).

| Cell populations | Markers |
| --- | --- |
| CD4^+^ T cells | CD3^+^ CD4^+^ CD8^-^ (Excluding NKT, γδ T, and MAIT cells) |
| CD8^+^ T cells | CD3^+^ CD8^+^ CD4^-^ (Excluding NKT, γδ T, and MAIT cells) |
| Double-negative (DN) T cells | CD3^+^ CD4^-^ CD8^-^ (Excluding NKT, γδ T, and MAIT cells) |
| CD3^+^CD161^+^ T cells | CD3^+^ CD161^+^ (Excluding NKT, γδ T, and MAIT cells) |
| CD4^+^ naive T cells | CD4^+^ T cells CCR7^+^ CD45RA^+^ |
| CD4^+^ T_CM_ cells | CD4^+^ T cells CCR7^+^ CD45RA^-^ |
| CD4^+^ T_EM_ cells | CD4^+^ T cells CCR7^-^ CD45RA^-^ |
| CD4^+^ T_EMRA_ cells | CD4^+^ T cells CCR7^-^ CD45RA^+^ |
| CD8^+^ naïve T cells | CD8^+^ T cells CCR7^+^ CD45RA^+^ |
| CD8^+^ T_CM_ cells | CD8^+^ T cells CCR7^+^ CD45RA^-^ |
| CD8^+^ T_EM_ cells | CD8^+^ T cells CCR7^-^ CD45RA^-^ |
| CD8^+^ T_EMRA_ cells | CD8^+^ T cells CCR7^-^ CD45RA^+^ |
| Vδ2^+^ γδ T cells | CD3^+^ TCRγδ^+^ TCRVδ2^+^ (Excluding NKT cells) |
| Vδ2^-^ γδ T cells | CD3^+^ TCRγδ^+^ TCRVδ2^-^ (Excluding NKT cells) |
| MAIT cells | CD3^+^ CD161^+^ TCRVα7.2^+^ (Excluding NKT and γδ T cells) |
| NKT cells | CD3^+^ Vα24JαQ TCR^+^ |
| CD56^dim^ NK cells | CD3^-^ CD19^-^ CD20^-^ CD14^-^ CD56^dim^ |
| CD56^bright^ NK cells | CD3^-^ CD19^-^ CD20^-^ CD14^-^ CD56^bright^ |
| Classical monocytes | CD3^-^ CD19^-^ CD20^-^ CD56^-^ HLADR^+^ CD14^+^ CD16^-^ |
| Intermediate monocytes | CD3^-^ CD19^-^ CD20^-^ CD56^-^ HLADR^+^ CD14^+^ CD16^+^ |
| Non-classical monocytes | CD3^-^ CD19^-^ CD20^-^ CD56^-^ HLADR^+^ CD14^-^ CD16^+^ |
| Dendritic cells (DCs) | CD3^-^ CD19^-^ CD20^-^ CD56^-^ HLADR^+^ CD14^-^ CD16^-^ CD11c^+^ CD123^+^ |
| Myeloid DCs (mDCs) | Dendritic cells CD11c^+^ CD123^-^ |
| Plasmacytoid DCs (pDCs) | Dendritic cells CD11c^-^ CD123^+^ |
| Innate Lymphoid Cells-1 (ILC1) | CD3^-^ CD19^-^ CD14^-^ CD16^-^ CD27^+^ CD56^+^/^-^ CD161^+^ c-Kit^-^ CRTH2^-^ |
| Innate Lymphoid Cells-2 (ILC2) | CD3^-^ CD19^-^ CD14^-^ CD16^-^ CD27^+^ CD56^+^/^-^ CD161^+^ c-Kit^+^/^-^CRTH2^+^ |
| Innate Lymphoid Cells-3 (ILC3) | CD3^-^ CD19^-^ CD14^-^ CD16^-^ CD27^+^ CD56^+^/^-^ CD161^+^ c-Kit^+^ CRTH2^-^ |

**Supplementary table 3**: 40 colour antibody panel for immune profiling of cord blood mononuclear cells (CBMCs) and peripheral blood mononuclear cells (PBMCs).

| Immunogen | Conjugate | Manufacturer | Clone |  |
| --- | --- | --- | --- | --- |
| Antibody stain- step 1 at room temperature | | | |  |
| CD297 (PD-1) | BUV615 | BD Biosciences | EH12.1 |  |
| CD117 (c-Kit) | Alexa Fluor 647 | BioLegend | 104-D2 |  |
| CD294 (CRTH2) | BV510 | BD Biosciences | BM16 |  |
| CD194 (CCR4) | BV605 | BioLegend | L291H4 |  |
| CD196 (CCR6) | BV650 | BD Biosciences | 11A9 |  |
| CD197 (CCR7) | PE/Fire810 | BioLegend | G043H7 |  |
| CD183 (CXCR3) | APC | BD Biosciences | 1C6 |  |
| CD185 (CXCR5) | BUV805 | BD Biosciences | RF8B2 |  |
| CD27 | PerCP-eFluor 710 | BD Biosciences | 0323 |  |
| Antibody stain- step 2 on ice | | | | |
| CD3 | BUV395 | BD Biosciences | UCHT1 |  |
| CD4 | BUV661 | BD Biosciences | SK3 |  |
| CD28 | BUV563 | BD Biosciences | CD28.2 |  |
| CD38 | BUV496 | BD Biosciences | HIT2 |  |
| CD69 | BUV737 | BD Biosciences | FN50 |  |
| CD56 | BV570 | BioLegend | HCD56 |  |
| CD57 | Pacific Blue | BioLegend | HNK-1 |  |
| CD141 | BV750 | BD Biosciences | 1A4 |  |
| HLADR | BV421 | BioLegend | L243 |  |
| Vα7.2 TCR | BV711 | BioLegend | 3C10 |  |
| Vδ2 | BV480 | BD Biosciences | B6 |  |
| γδ TCR | FITC | BD Biosciences | 11F2 |  |
| IgM | Spark blue 550 | BioLegend | MHM-88 |  |
| iNKT 6B11  (Vα24Jα18) | BV785 | BioLegend | 6B11 |  |
| CD11c | PerCP | BioLegend | Bu15 |  |
| CD45RA | PerCP/Cy5.5 | BD Biosciences | HI100 |  |
| IgD | PerCP/Fire806 | BioLegend | W18340F |  |
| CD159a (NKG2A) | PE | Beckman Coulter | Z199 |  |
| CD24 | PE-AF610 | BD Biosciences | SN3 |  |
| CD1c | PECy5 | BioLegend | L161 |  |
| CD16 | BYG710 | Cytek Biosciences | 3GB |  |
| CD161 | PEvio770 | Miltenyi Biotec | 191 B8 |  |
| CD8 | APC | BioLegend | SK1 |  |
| CD19 | Spark NIR 685 | BioLegend | HIB19 |  |
| CD127 | APC-R700 | BD Biosciences | HIL-7R-M21 |  |
| IgG | APC-H7 | BD Biosciences | G18-145 |  |
| Antibody stain- step 3 on ice | | | |  |
| CD14 | BB700 | BD Biosciences | M5E2 |  |
| CD20 | Pacific orange | Invitrogen | HI47 |  |
| CD25 | PE-CF594 | BD Biosciences | M-A251 |  |
| CD123 | RY586 | BD Biosciences | 7G3 |  |
| Zombie | NIR | BioLegend | - |  |


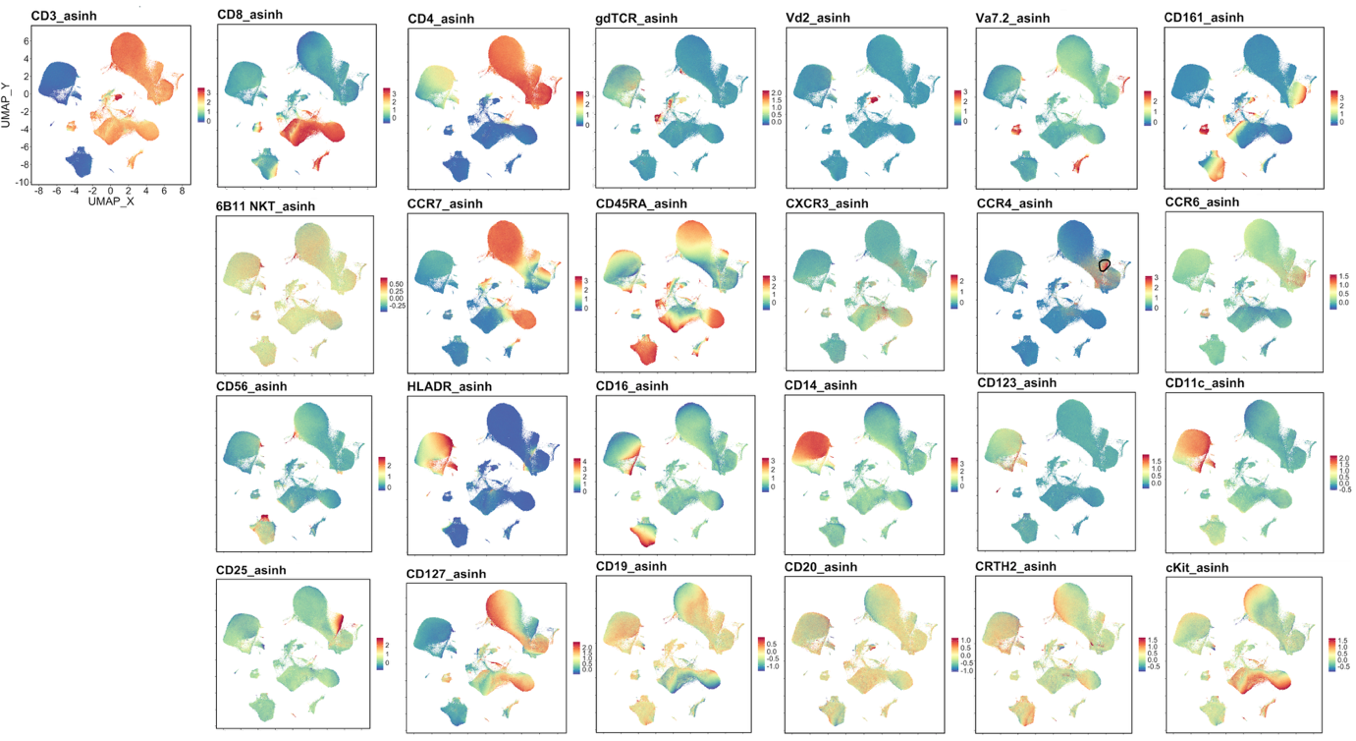


Supplementary figure 1. A combination of 24 samples from cord blood, young adults, and old adults peripheral blood was used to generate UMAP plots to display the expression levels of CD3, CD4, CD8, CD11c, CD14, CD16, CD19, CD20, CD25, CD56, CD123, CD127, CD161, CCR4, CCR6, CCR7, CXCR3, CD45RA, HLADR, γδ TCR, Vδ2 TCR, Vα7.2 TCR, Vα24Jα18 (6B11 NKT), CRTH2 and c-Kit to phenotypically characterise various subsets of innate-like T cells, CD4^+^ T cells, CD8^+^ T cells, ILCs, NK cells, monocytes and dendritic cells. A blue-green-red colour scale was applied to show the results. UMAP analysis was conducted using 750 iterations with a perplexity of 200.


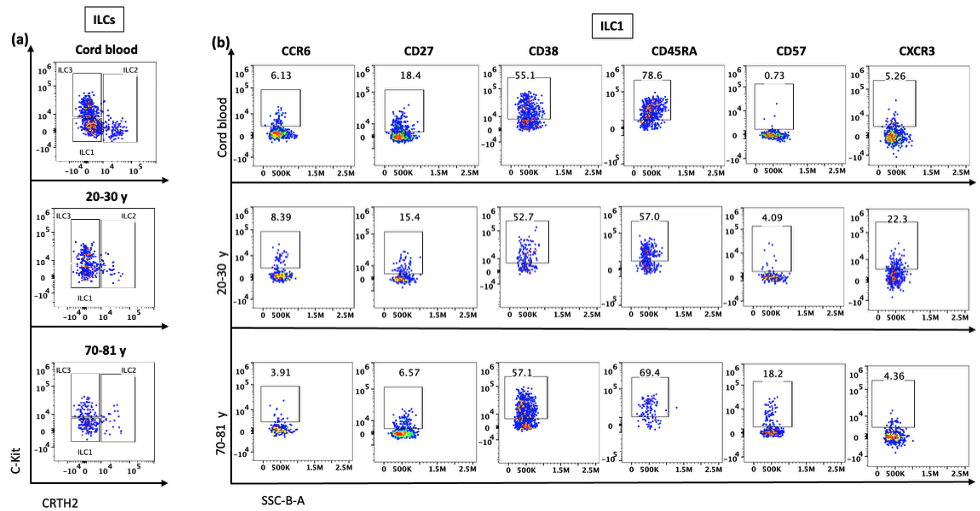

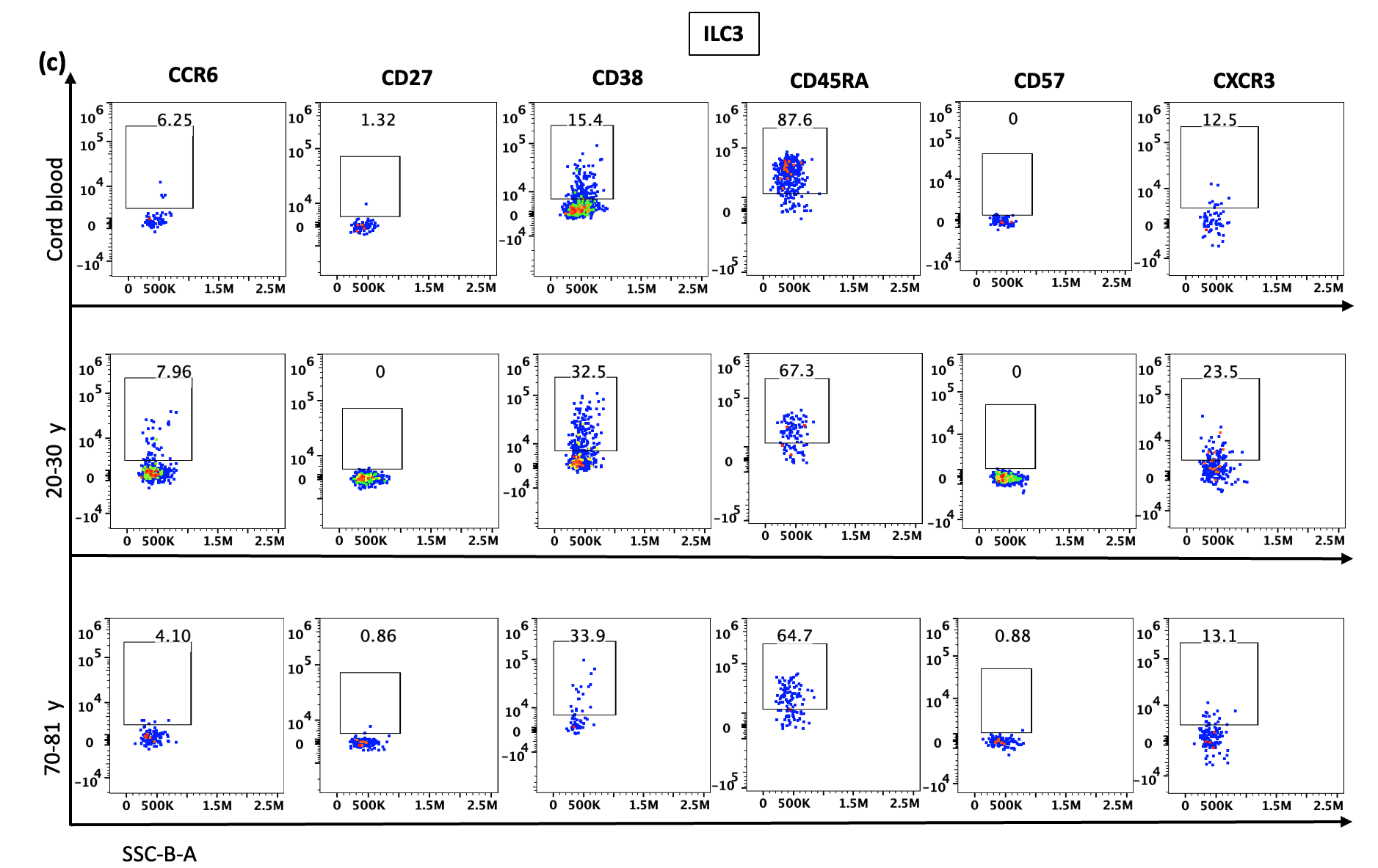


Supplementary figure 2. **The phenotype of Innate Lymphoid Cells (ILCs) changed with age. (a)** Flow cytometry plots from one individual of each age group representing the proportion of ILC1, 2 and 3 within: cord blood, 20-30 years old, and 70-81 years old. **(b, c)** Flow cytometry plots from one study participants per age group showing the expression levels of CCR6, CD27, CD38, CD45RA, CD57 and CXCR3 on **(b)** ILC1 and **(c)** ILC3 within the three age groups.


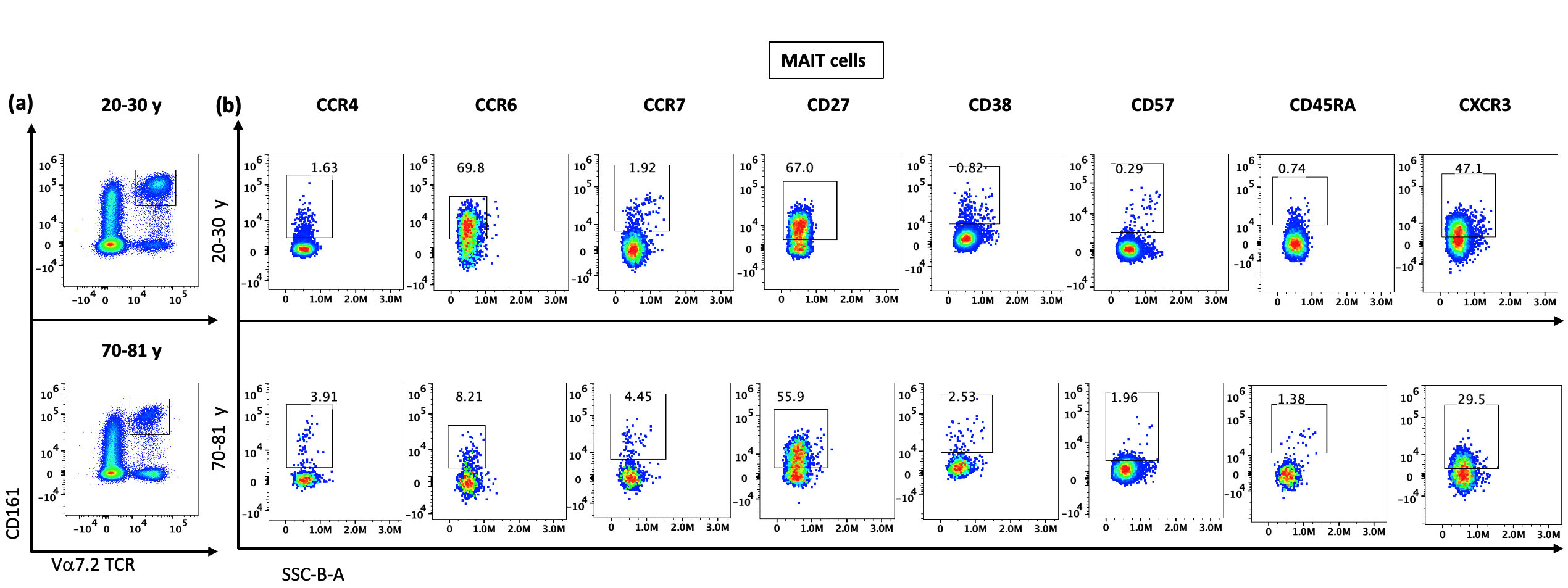


Supplementary figure 3. **The phenotype of MAIT cells changed with age.** **(a)** Flow cytometry plots of one individual from each adult age group showing the proportions of MAIT cells within the two age groups: 20-30 years old and 70-81 years old. **(b) Representative** flow cytometry plots from one of the study participants per age group show the expression of CCR4, CCR6, CCR7, CD27, CD38, CD57, CD45RA and CXCR3 on MAIT cells within the two age groups.


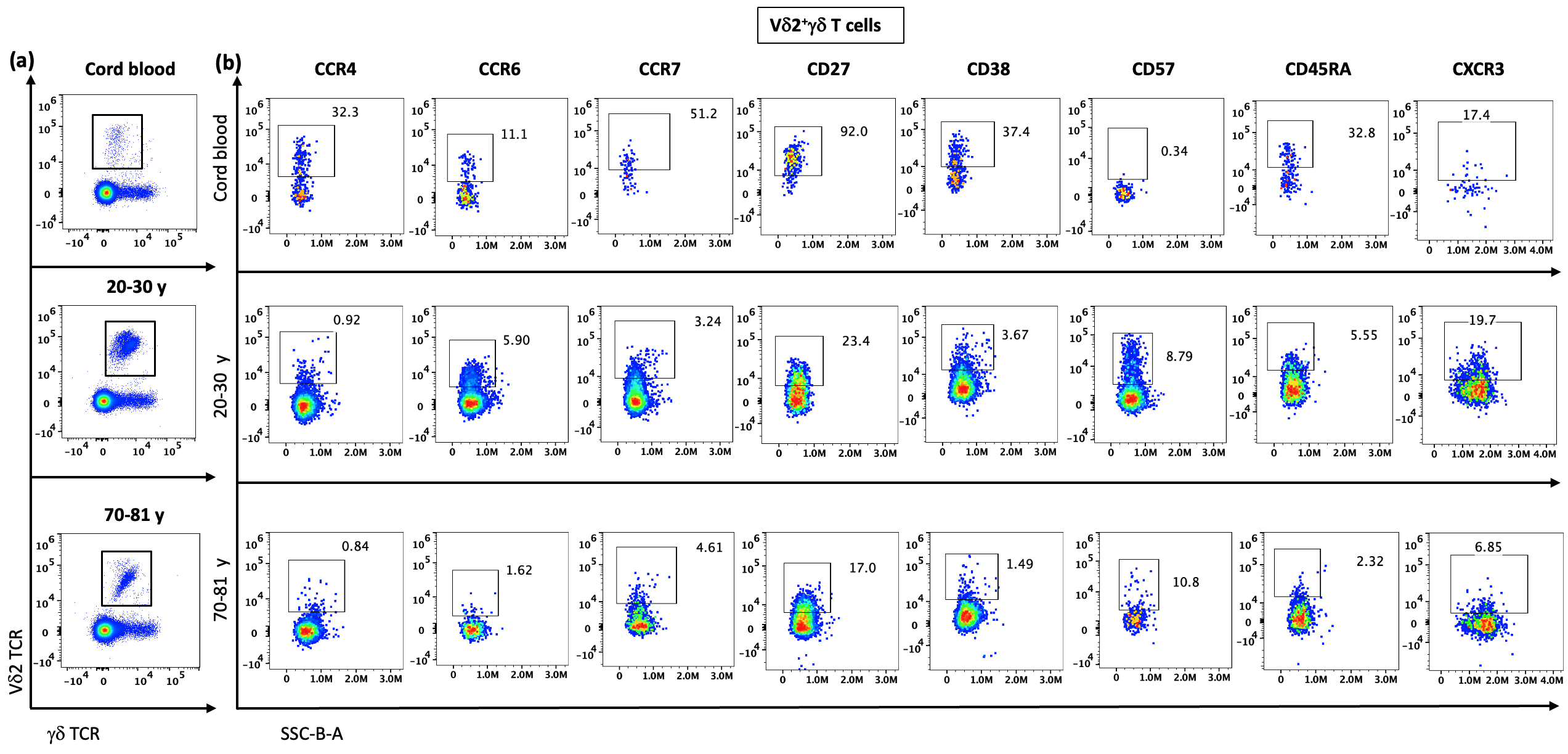


Supplementary figure 4. **The phenotype of Vδ2^+^ γδ T cells changed with age.** **(a)** Flow cytometry plots of one individual from each age group to show the proportions of Vδ2^+^ γδ T cells within the three age groups: cord blood, 20-30 years old, and 70-81 years old. **(b)** Flow cytometry plots of one study participants per age group to show the expression levels of CCR4, CCR6, CCR7, CD27, CD38, CD57, CD45RA and CXCR3 on Vδ2^+^ γδ T cells within the three age groups.


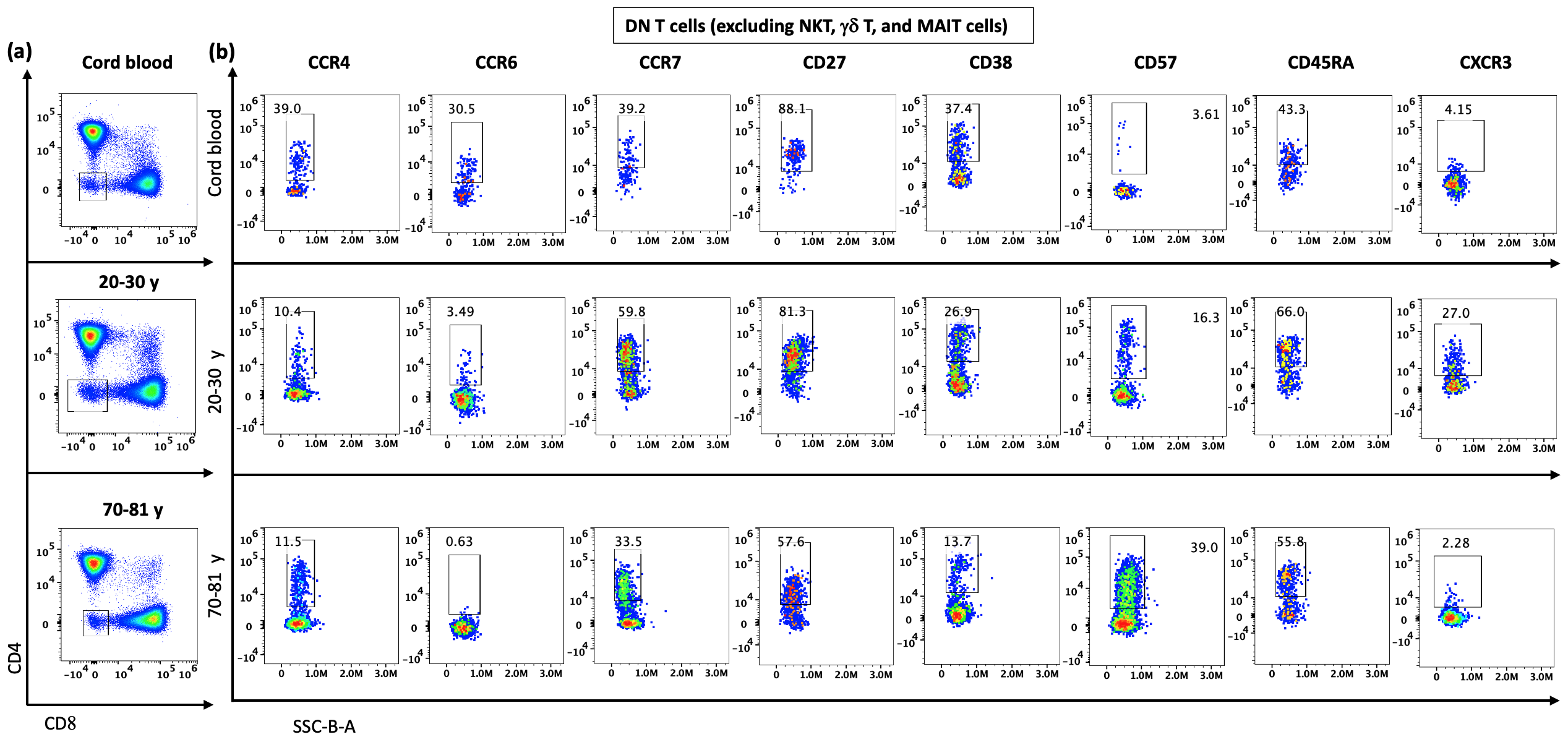


Supplementary figure 5. **The phenotype of Double-negative (DN) T cells changed with age.** **(a)** Flow cytometry plots of one individual per age group to show the proportions of DN T cells within the three age groups: cord blood, 20-30 years old, and 70-81 years old. **(b)** Flow cytometry plots from one study participants per age group to show the expression levels of CCR4, CCR6, CCR7, CD27, CD38, CD57, CD45RA and CXCR3 on DN T cells within the three age groups.


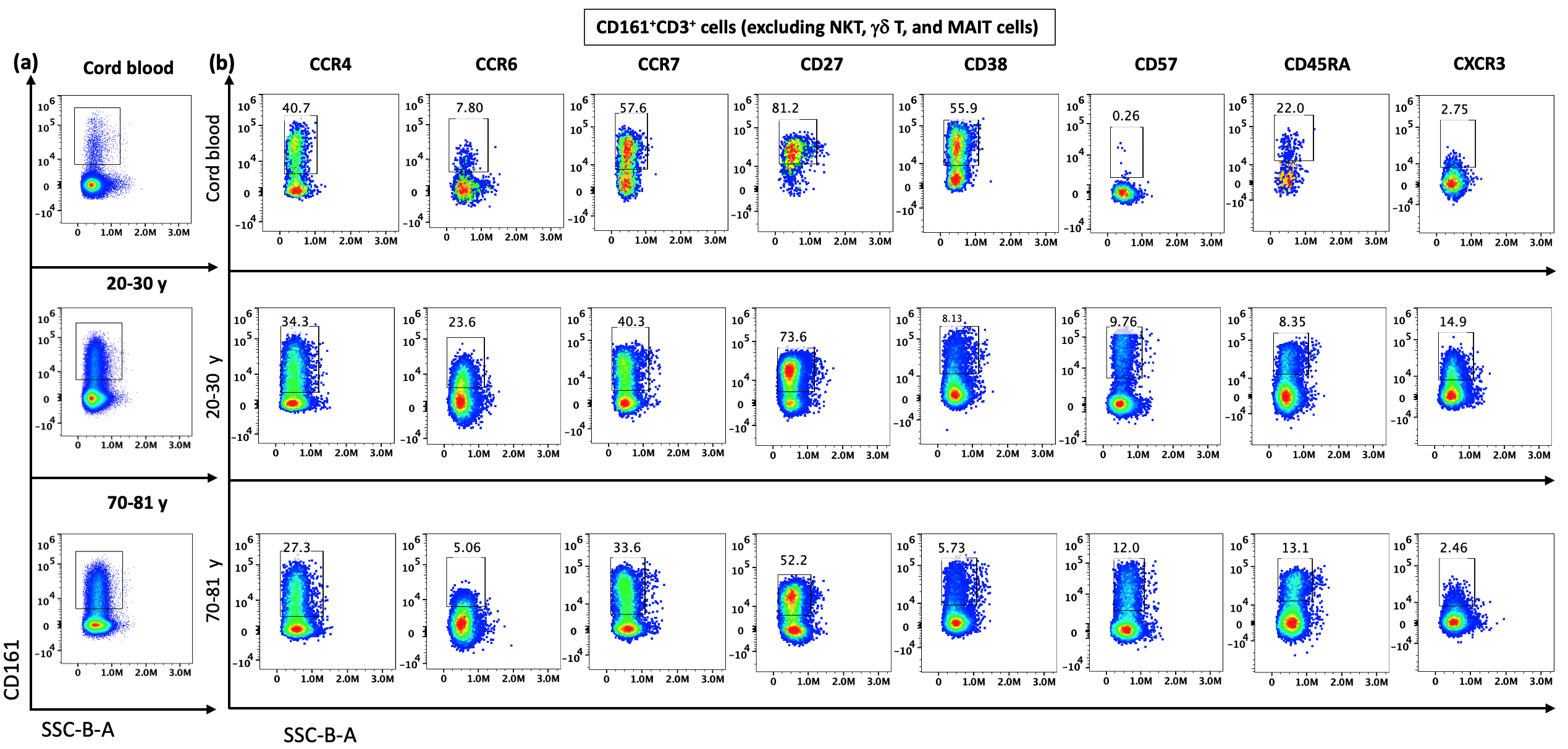


Supplementary figure 6. **The phenotype of CD3^+^CD161^int^ T cells changed with age. (a)** Flow cytometry plots of one individual per age group to show the proportions of CD3^+^CD161^int^ T cells within the three age groups: cord blood, 20-30 years old, and 70-81 years old. **(b)** Flow cytometry plots of one study participants per age group to show the expression levels of CCR4, CCR6, CCR7, CD27, CD38, CD57, CD45RA and CXCR3 on CD3^+^CD161^int^ T cells within the three age groups.

Supplementary figure 7. **The phenotype of Vδ2^-^ γδ T cells changed with age.** **(a)** Flow cytometry plots of one donor per age group to show the proportions of Vδ2^-^ γδ T cells within the three age groups: cord blood, 20-30 years old, and 70-81 years old. **(b)** Flow cytometry plots of one donor per age group to show the expression levels of CCR4, CCR7, CD27, CD38, CD57 and CXCR3 on Vδ2^-^ γδ T cells within the three age groups. **(c)** Violin plots show the age-related percentage of CCR4^+^, CCR7^+^, CD27^+^, CD38^+^, CD57^+^ and CXCR3^+^ Vδ2^-^ γδ T cells in cord blood (n = 8, orange), young adult blood (n = 8, blue) and older adult blood (n = 8, pink). Data are shown with the median. Each dot represents data from one donor, and each colour represents one age group. A nonparametric Kruskal–Wallis test with Dunn’s multiple comparisons test was used to compare all three groups. *P*-values are *P* > 0.05 (ns), **P* ≤ 0.05; ***P* ≤ 0.01; ****P* ≤ 0.001; *****P* ≤ 0.0001.


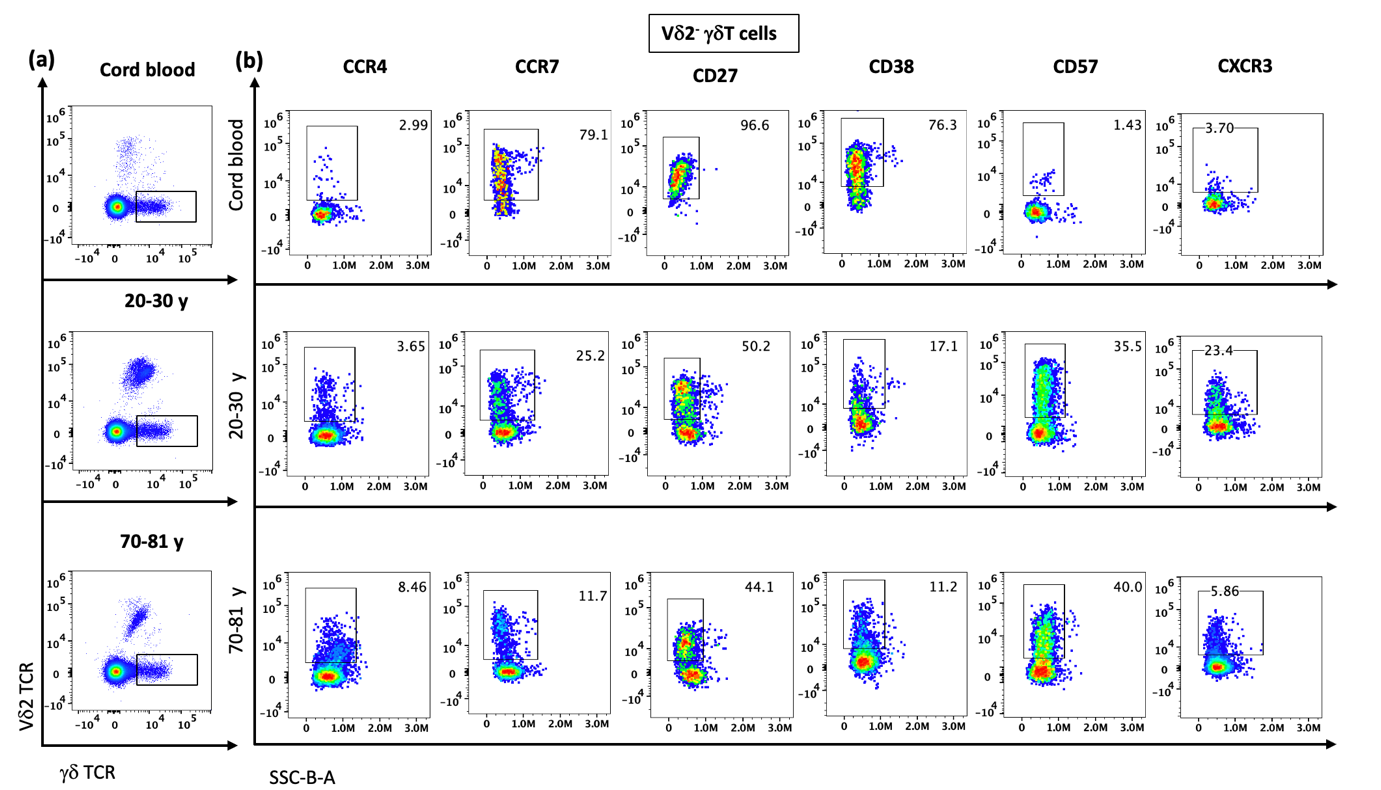

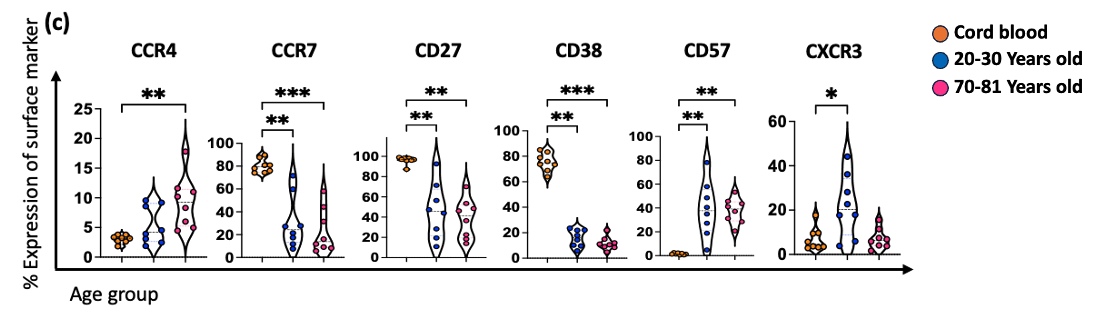

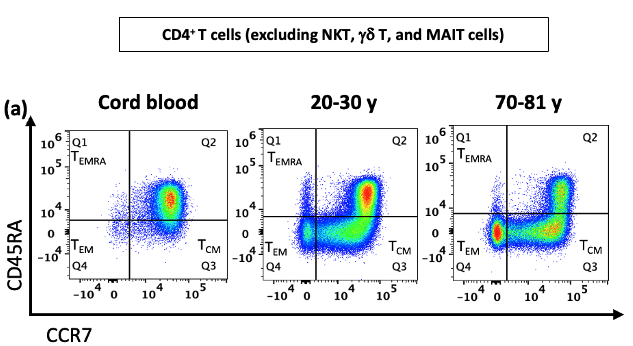

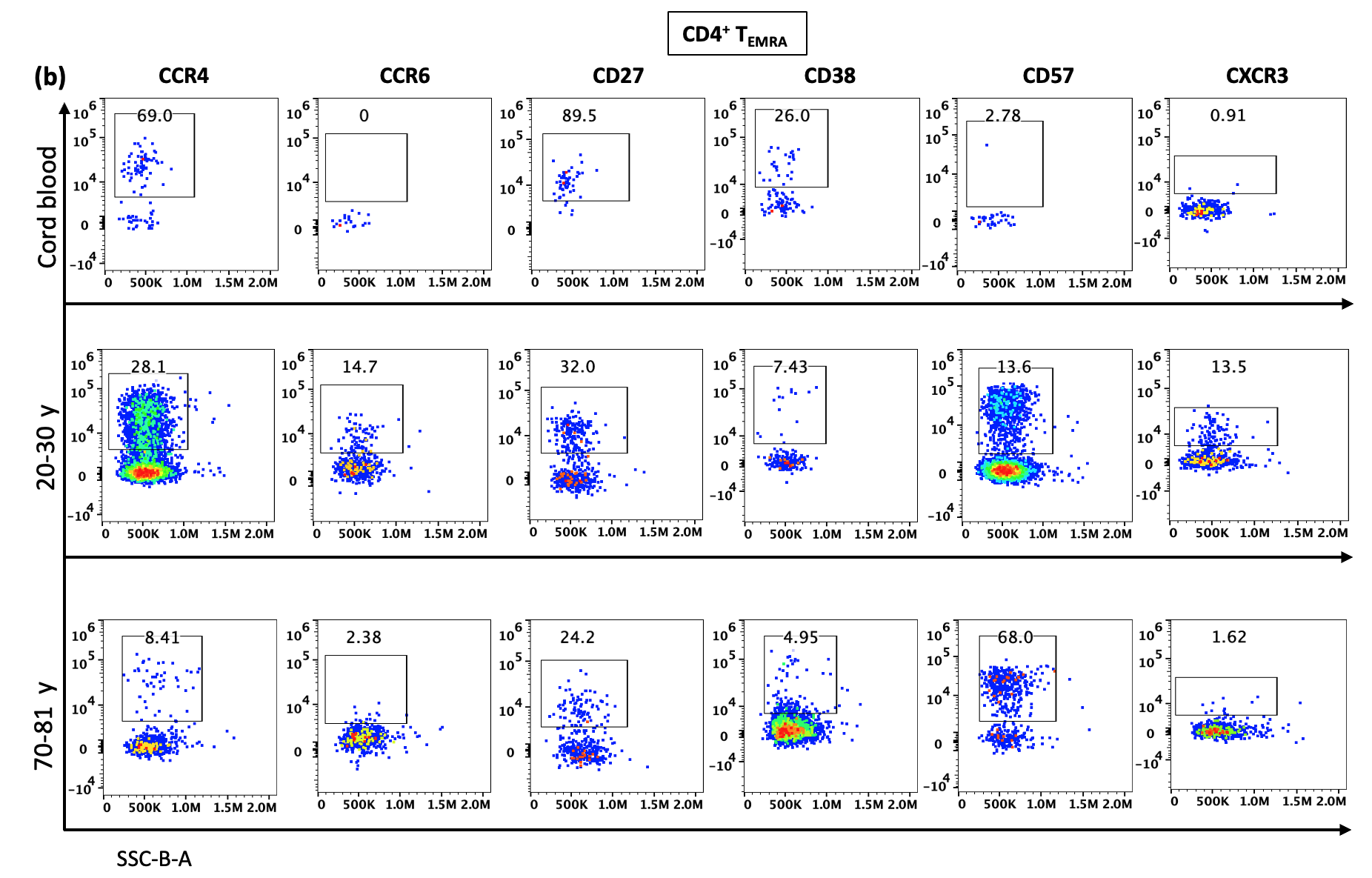

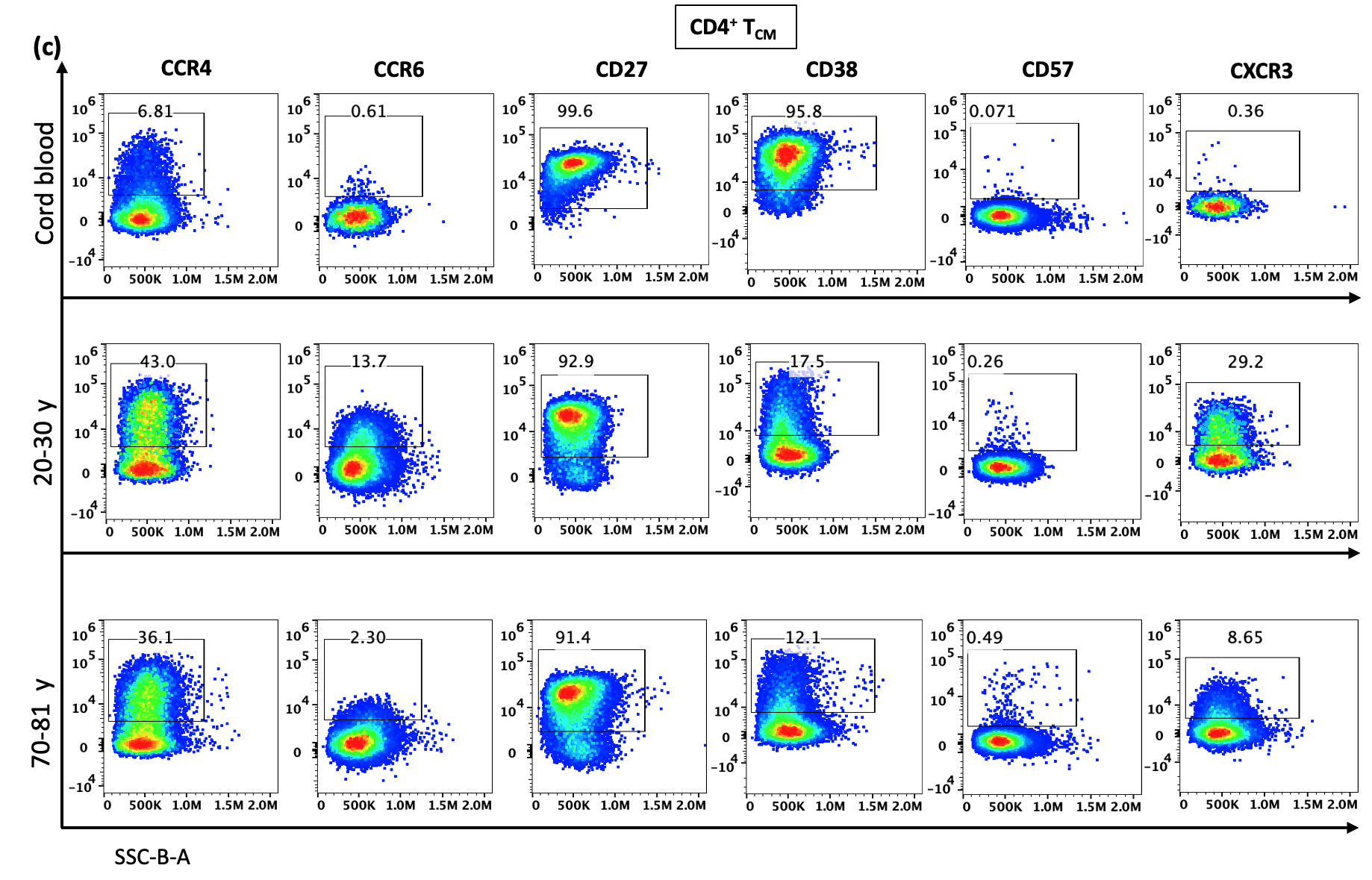


**
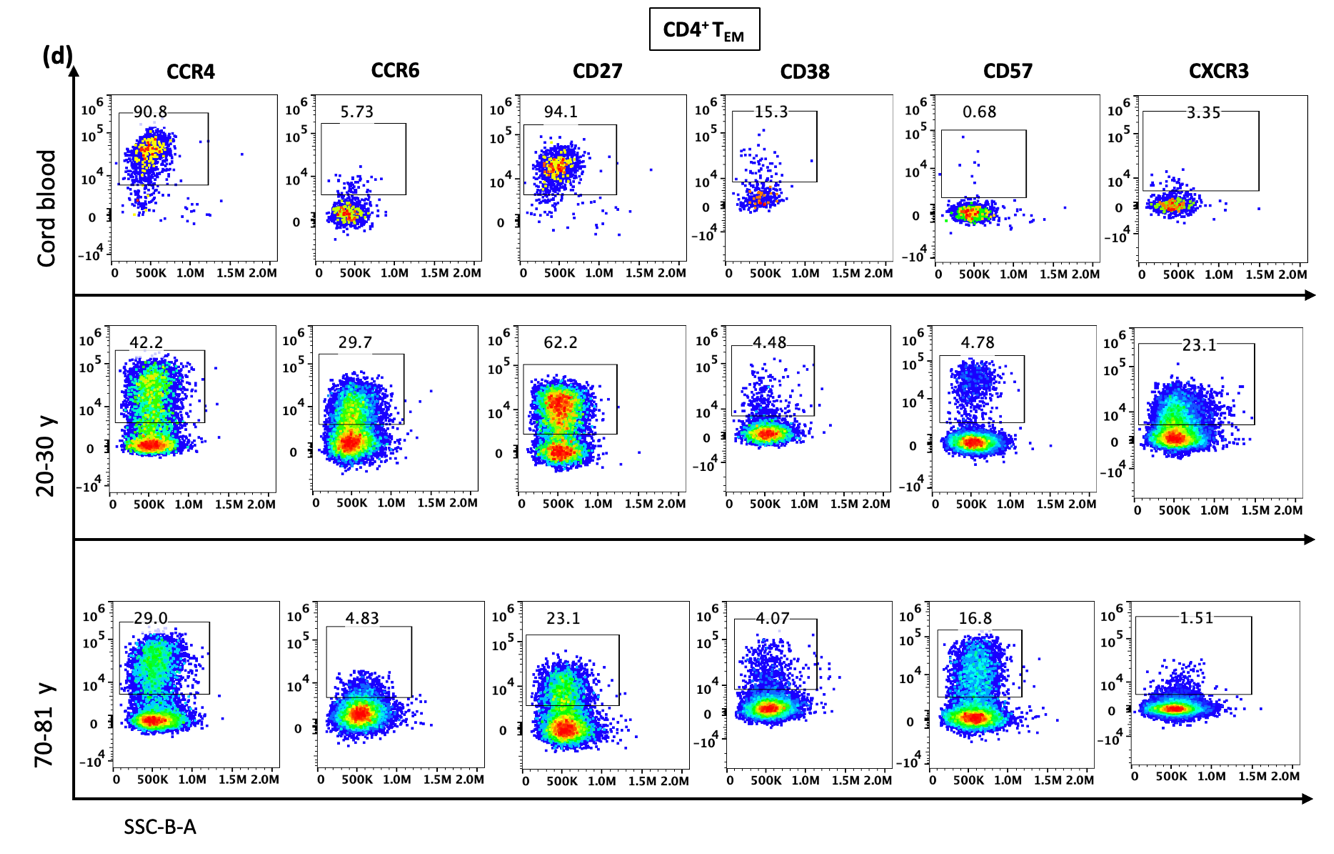
**

Supplementary figure 8. **The phenotype of memory populations of CD4^+^ T cells changed with age.** **(a)** Flow cytometry plots of one individual per age group to show the proportions of T central memory (T_CM_), T effector memory (T_EM_) and T effector memory CD45RA^+^ (T_EMRA_) CD4^+^ T cells within the three age groups: cord blood, 20-30 years old, and 70-81 years old. **(b-d)** Flow cytometry plots of one study participants per age group to show the expression levels of CCR4, CCR6, CD27, CD38, CD57 and CXCR3 on **(b)** T_EMRA_, **(c)** T_CM_ and **(d)** T_EM_ CD4^+^ T cells within the three age groups.


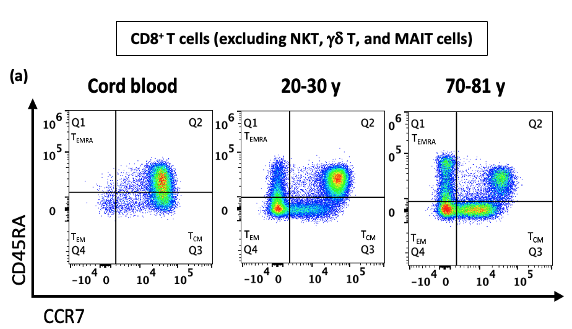

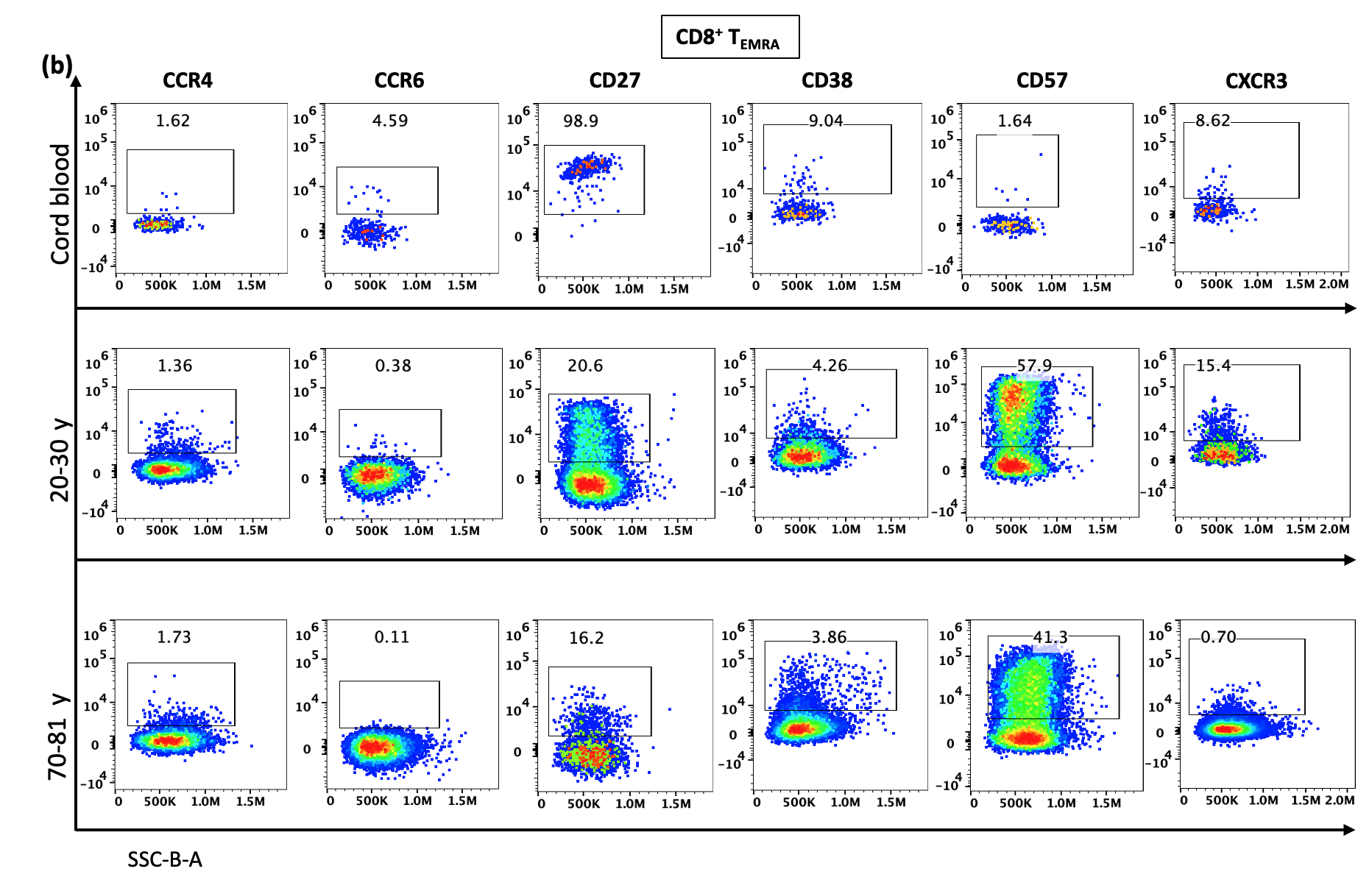

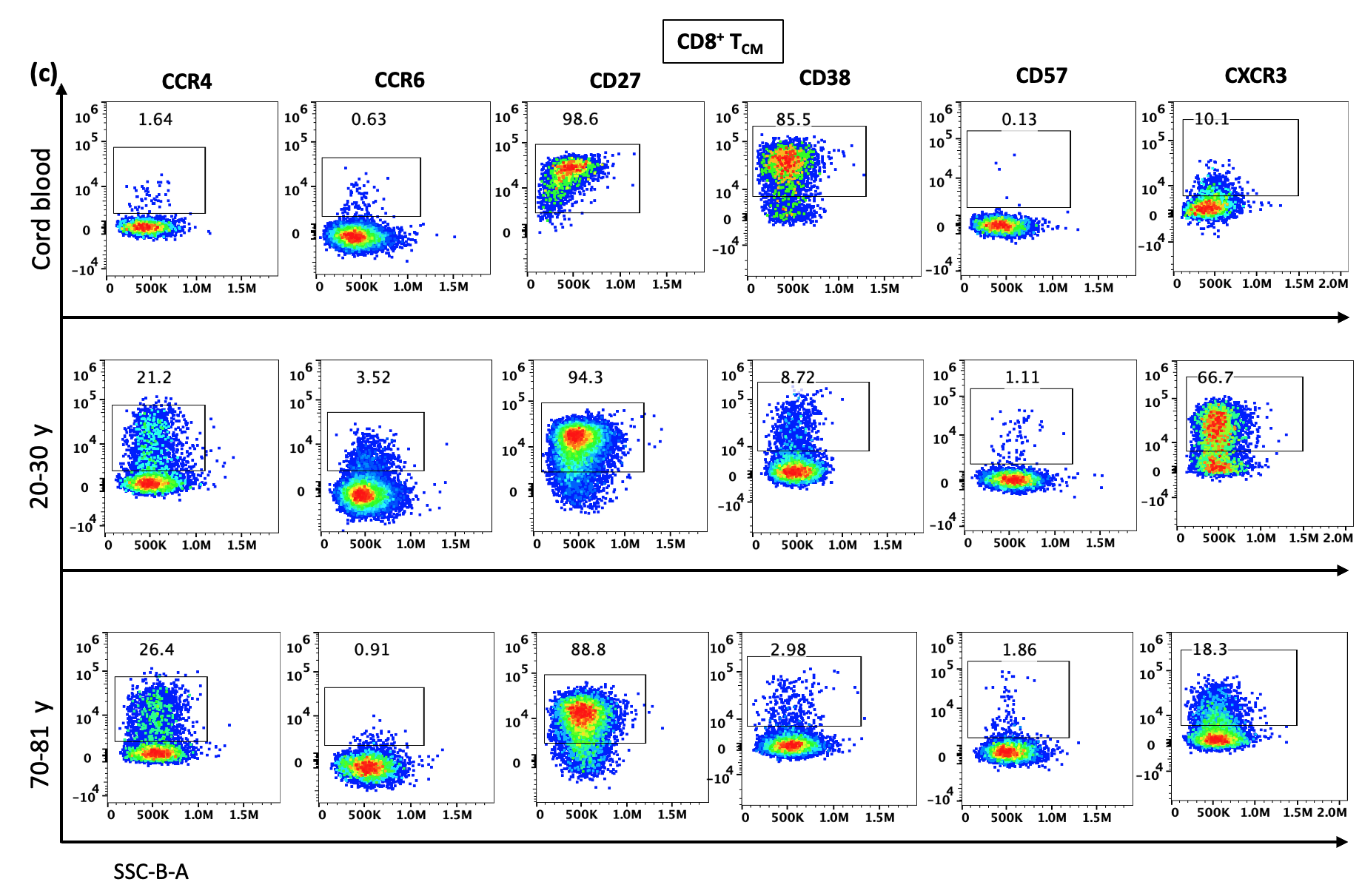


**
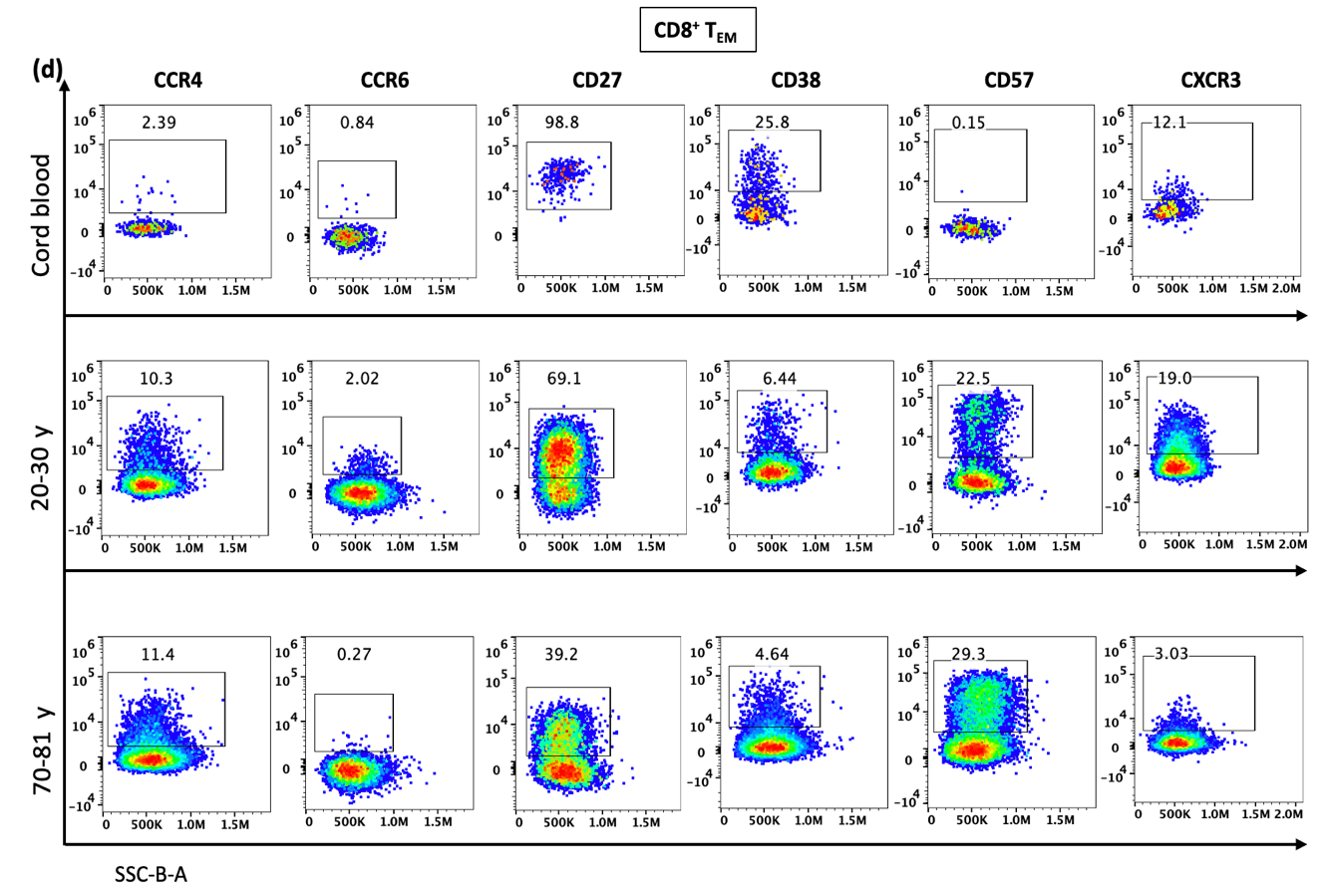
**

Supplementary figure 9. **The phenotype of three memory populations of CD8^+^ T cells changed with age.** **(a)** Flow cytometry plots of one individual per age group to show the proportions of T central memory (T_CM_), T effector memory (T_EM_) and T effector memory CD45RA^+^ (T_EMRA_) CD8^+^ T cells within the three age groups: cord blood, 20-30 years old, and 70-81 years old. **(b-d)** Flow cytometry plots of one study participants per age group to show the expression levels of CCR4, CCR6, CD27, CD38, CD57 and CXCR3 on **(b)** T_EMRA_, **(c)** T_CM_ and **(d)** T_EM_ CD8^+^ T cells within the three age groups.


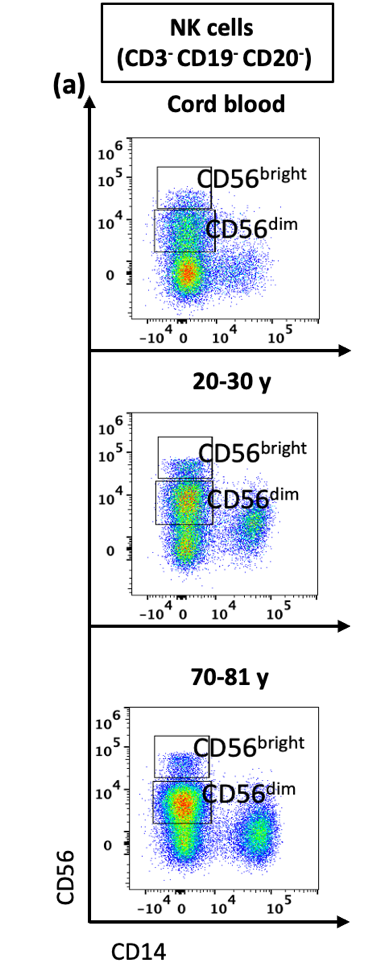

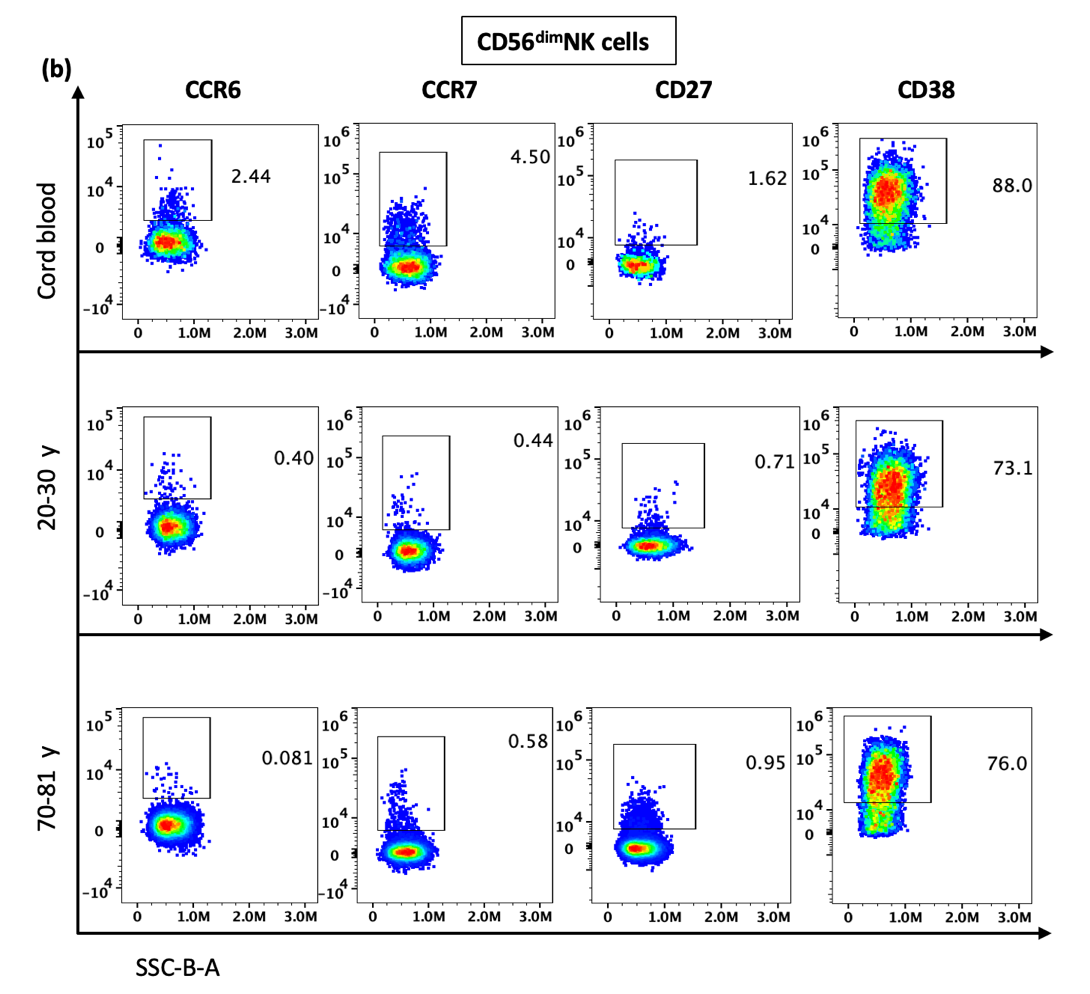


**
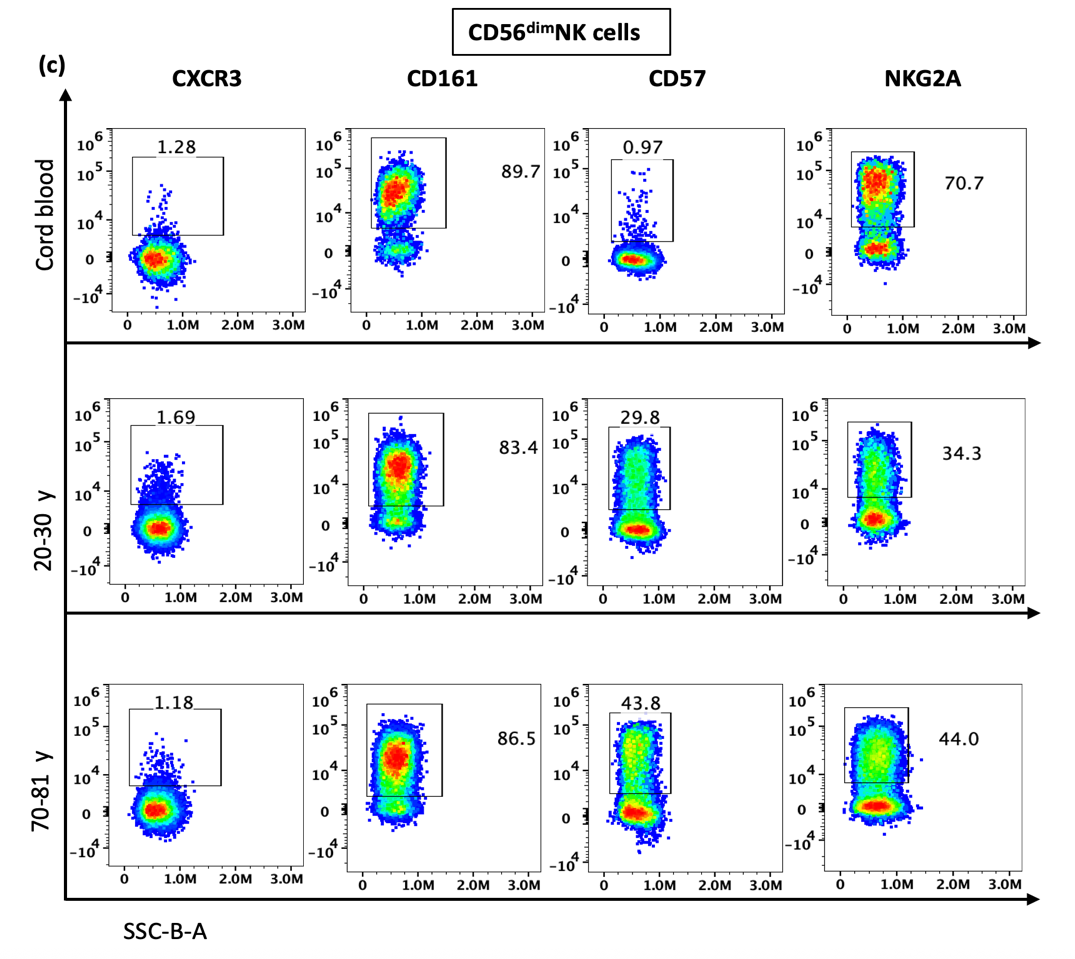
**


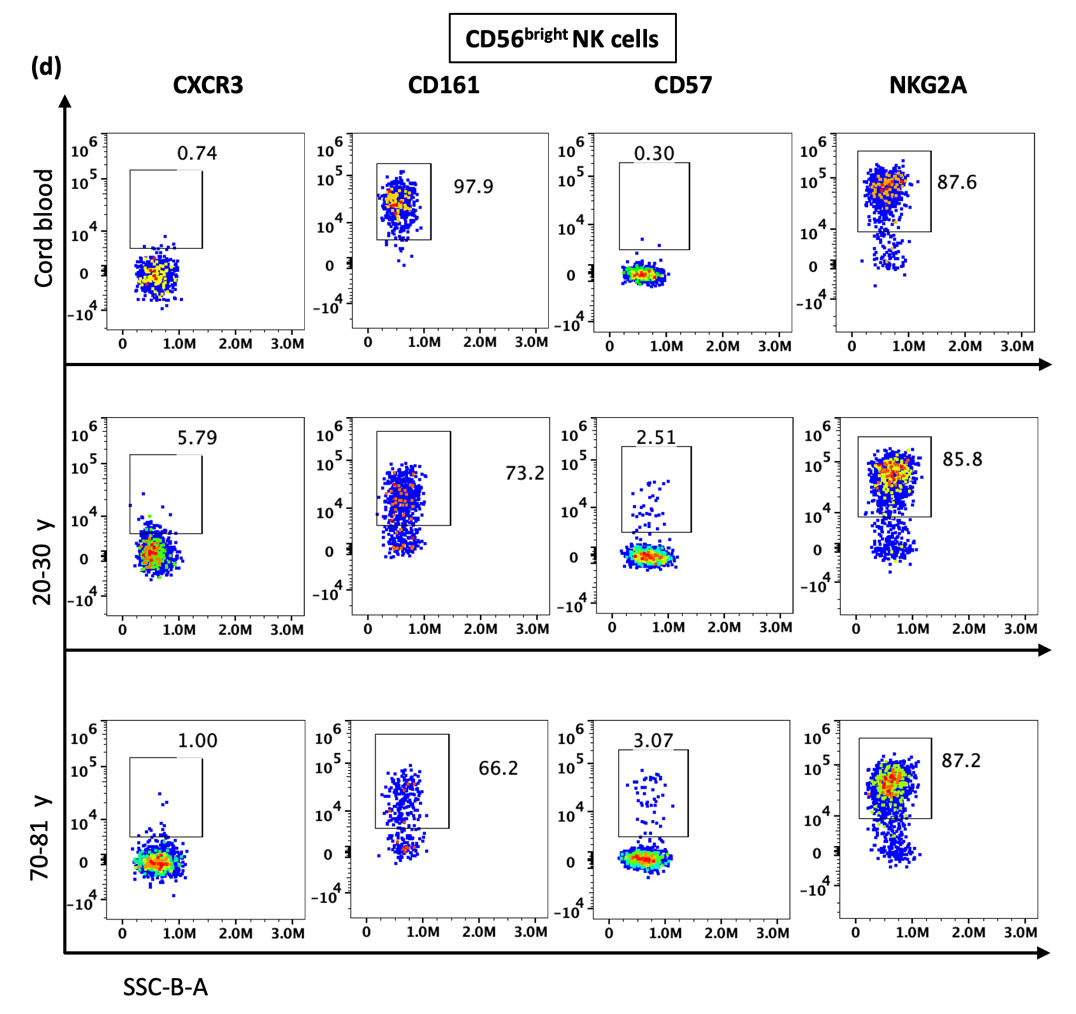

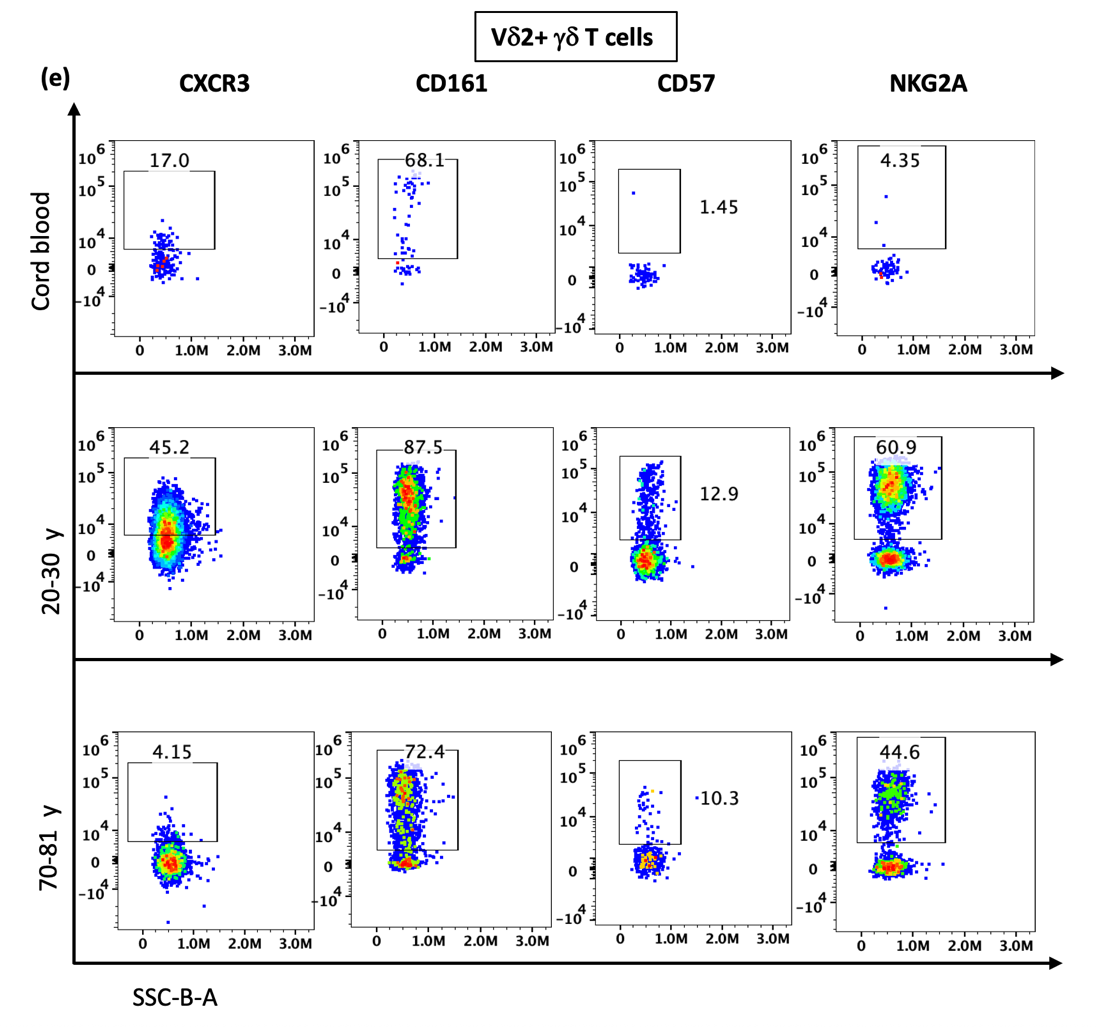


Supplementary figure 10. **The phenotype of CD56^dim^ NK cells and CD56^bright^ NK cells changed with age.** **(a)** Flow cytometry plots of one individual per age group to show the proportions of CD56^dim^ NK cells, and CD56^bright^ NK cells within the three age groups: cord blood, 20-30 years old, and 70-81 years old. **(b)** Flow cytometry plots of one study participants per age group to show the expression levels of CCR6, CCR7, CD27 and CD38 on CD56^dim^ NK cells. **(c-e)** Flow cytometry plots of one study participants per age group to show the expression levels of CXCR3, CD161, CD57, and NKG2A on **(c)** CD56^dim^ NK cells, **(d)** CD56^bright^ NK cells and **(e)** Vδ2^+^ γδ T. cells within the three age groups.


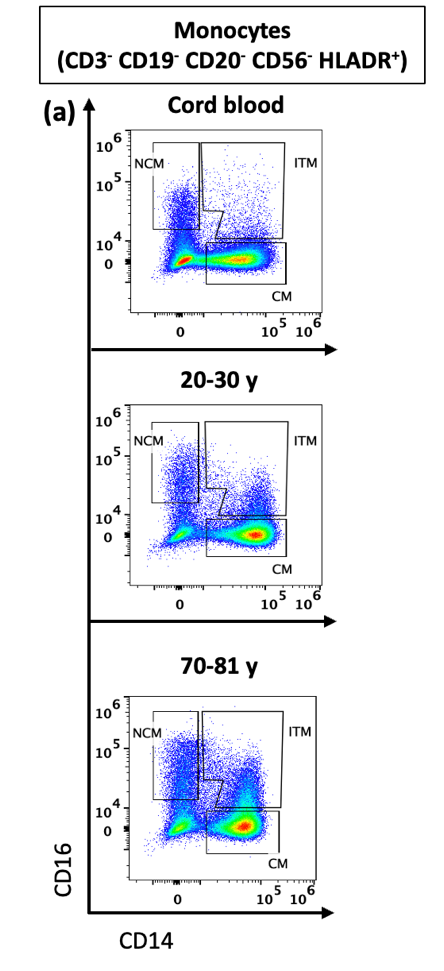

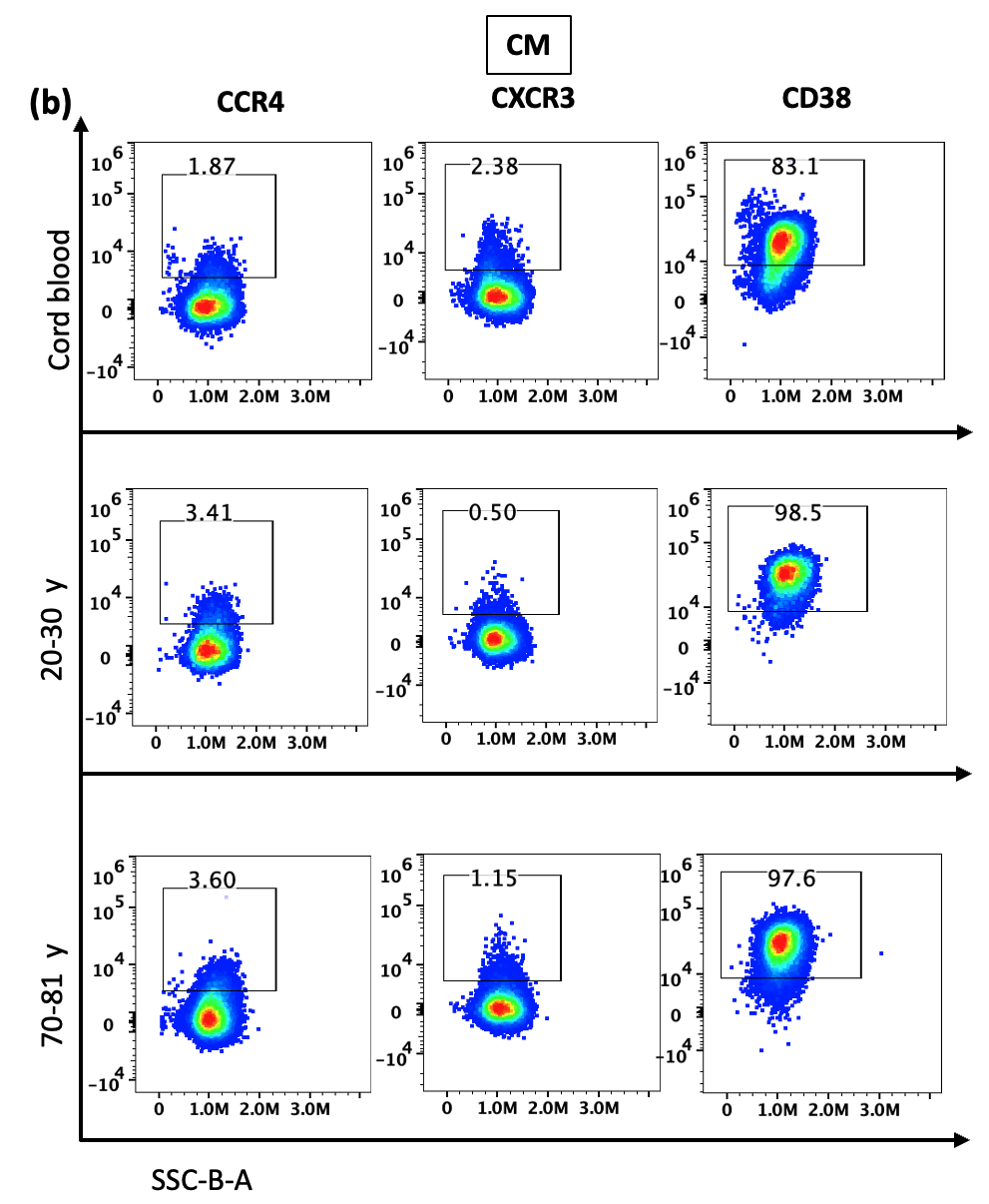

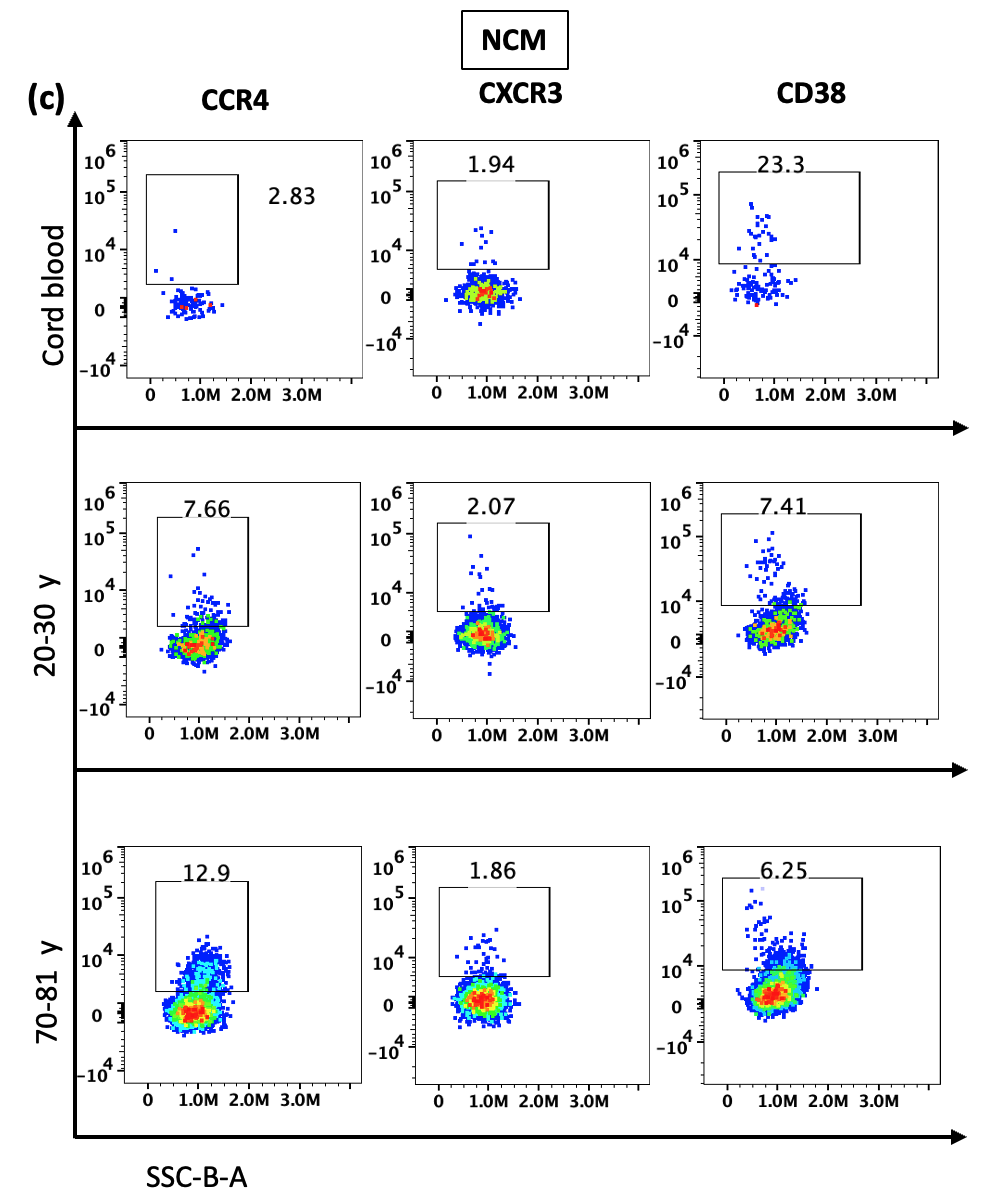

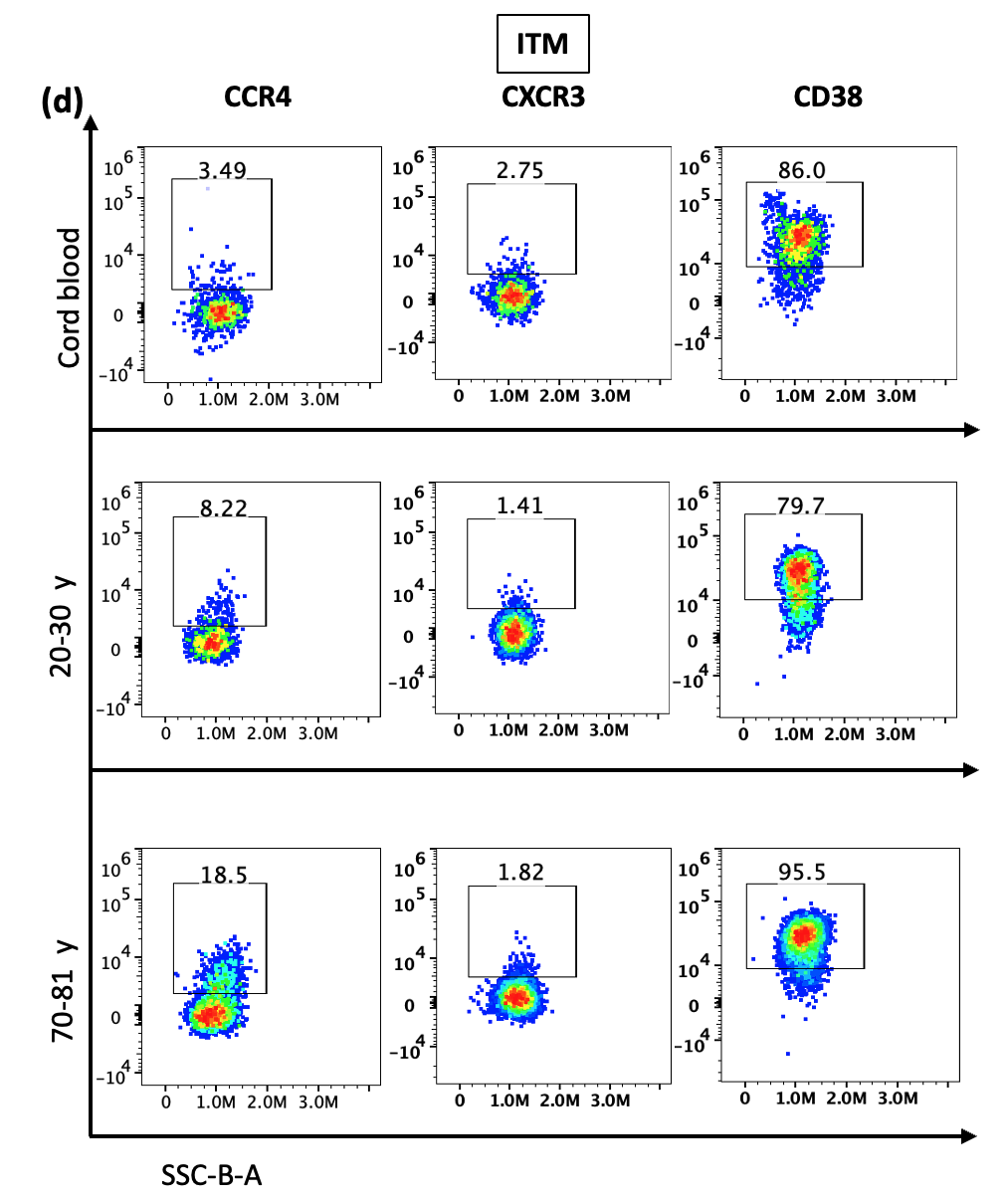


Supplementary figure 11. **The phenotype of monocyte subsets changed with age. (a)** The flow cytometry plots from one individual in each age group show the proportions of classical monocytes (CM), non-classical monocytes (NCM), and intermediate monocytes (ITM) within the three age groups: cord blood, 20-30 years old, and 70-81 years old. **(b-d)** Flow cytometry plots from one of the study participants per age group show the expression levels of CCR4, CXCR3, and CD38 on (**b)** CM, **(c)** NCM, and **(d)** ITM within the three age groups.

**
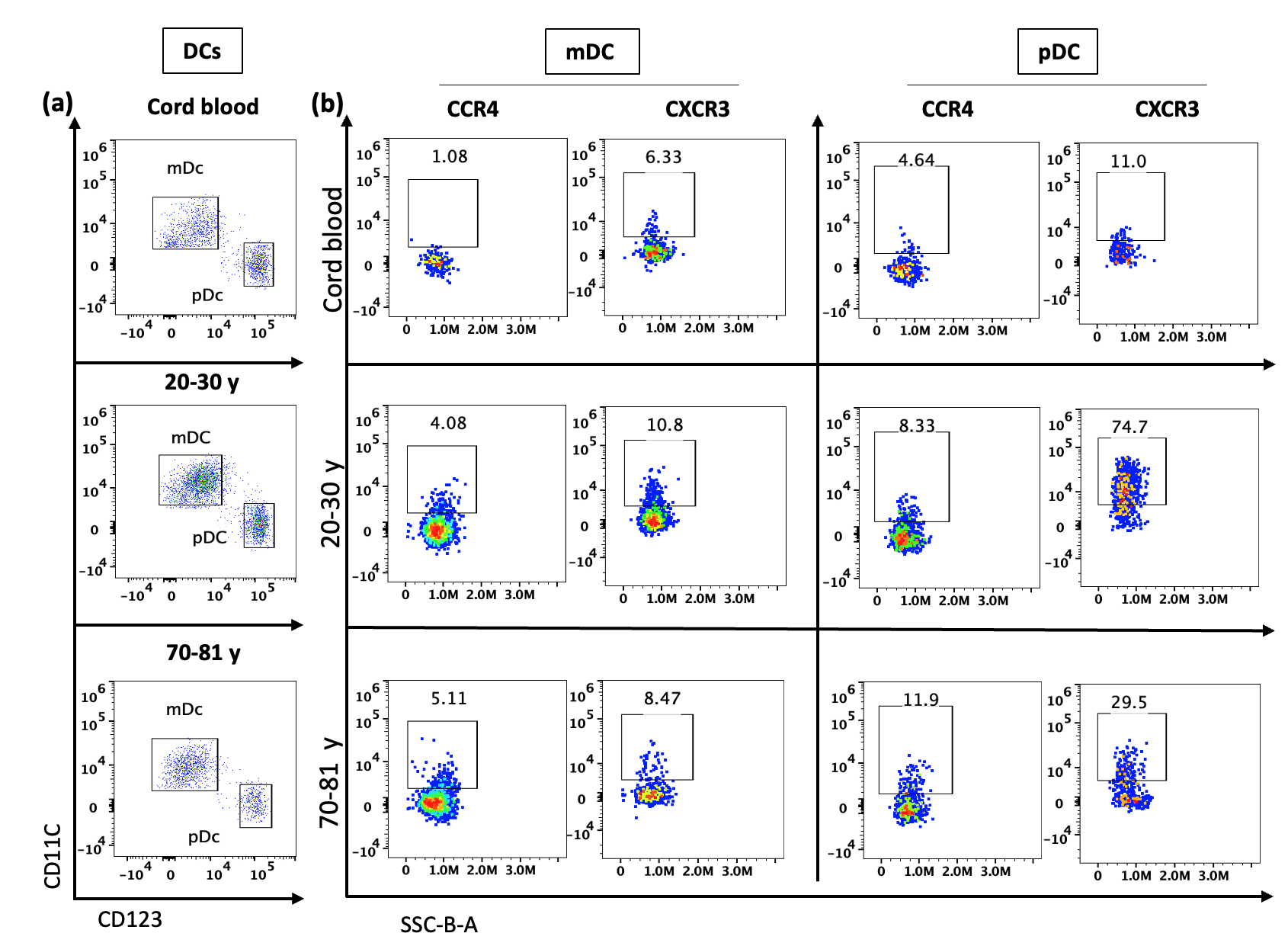
**

Supplementary figure 12. **The phenotype of dendritic cell subsets changed with age. (a)** Flow cytometry plots of one individual per age group to show the proportions of myeloid DCs (mDCs) and plasmacytoid DCs (pDCs) within the three age groups: cord blood, 20-30 years old, and 70-81 years old. **(b)** Flow cytometry plots of one participant per age group to show the expression levels of CCR4 and CXCR3 on mDCs and pDCs cells within the three age groups.


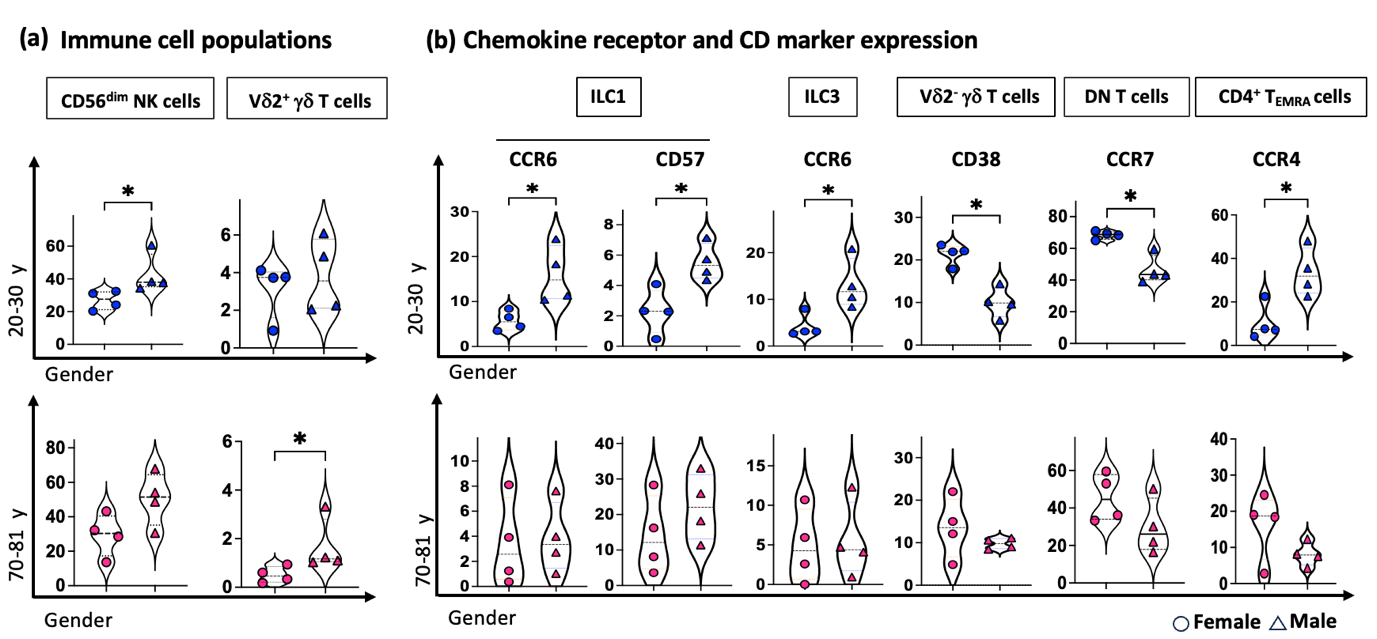


Supplementary figure 13. **Gender associated differences in immune cell composition and their phenotypes. (a)** The violin plots representing the frequency of CD56^dim^ NK cells and Vδ2⁺ γδ T cells within young (20–30 years old) (blue) and older (70–81 years old) (pink) adult groups between female (circle) and male (triangle). **(b)** The violin plots representing the percentage of CCD6^+^ and CD57^+^ ILC1, CCR6^+^ ILC3, CD38^+^ Vδ2⁻ γδ T cells, CCR7^+^ DN T cells, and CCR4^+^ CD4⁺ T_EMRA_ cells. The Mann Whitney *U*-test was used to compare the frequency of cells between females and males. Data is shown with median. Each dot represents one individual.
